# NF-κB-Inducing Kinase Is Essential for Effective c-Rel Transactivation and Binding to the *Il12b* Promoter in Macrophages

**DOI:** 10.3390/biology14010033

**Published:** 2025-01-03

**Authors:** Natalia Cuesta, Anna D. Staniszewska, Cristóbal Moreno, Carmen Punzón, Manuel Fresno

**Affiliations:** 1 Department of Cell Biology and Histology, School of Medicine, Universidad Complutense de Madrid, Avda Complutense s/n, 28040 Madrid, Spain; 2 Department of Biochemistry and Molecular Biology, Centro de Biología Molecular Severo Ochoa, Universidad Autónoma de Madrid—Consejo Superior de Investigaciones Científicas, Nicolás Cabrera 1, 28049 Madrid, Spaincmoreno.del@gmail.com (C.M.); cpunzon.cp@gmail.com (C.P.); mfresno@cbm.uam.es (M.F.)

**Keywords:** macrophages, NIK, IL-12, c-Rel

## Abstract

This study investigates the role of NIK in activating specific inflammatory genes in macrophages, focusing on the effect of a mutation in NIK found in alymphoplasia (*aly*/*aly*) mice. Mouse peritoneal macrophages from *aly*/*aly* mice showed a severe defect in the production of some pro-inflammatory cytokines, such as IL-12. This effect seemed to take place at the transcriptional level, as shown by the reduced transcription of *Il12b* and *Il12a* in *aly*/*aly* macrophages after exposure to the TLR4 agonist LPS. Immunoprecipitation studies showed that the binding of NIK to c-Rel was not efficient in RAW 264.7 cells over-expressing the *aly*/*aly* mutation. In addition, the shuttling of c-Rel to the nucleus was shown to be impaired in *aly*/*aly* macrophages in response to LPS. When looking more specifically at the regulation of the *Il12b* promoter, we found that c-Rel bound to the NF-kB consensus sequence in macrophages from WT mice 1 hr. after LPS challenge, whereas in *aly*/*aly* macrophages, the transcription factor bound to the promoter was p65. These findings indicate that NIK is essential for efficient c-Rel activation and proper inflammatory responses. NIK dysfunction could lead to weakened immune responses, and targeting this pathway may help in developing therapies for immune-related conditions.

## Simple Summary

This study explores how a mutation in NF-κB-inducing kinase (NIK) affects the production of pro-inflammatory molecules by macrophages. Normally, NIK helps activate a transcription factor called c-Rel, which regulates the expression of several genes. However, in mice with a mutation in NIK (*aly*/*aly* mice), macrophages produced lower levels of certain inflammatory molecules, such as Interleukin-12, after exposure to bacterial components. Mutated NIK hindered the ability of c-Rel to bind to DNA and enter the cell nucleus. Instead, another protein, p65, became more involved in binding but did not fully compensate for c-Rel’s role. These findings highlight the importance of NIK in managing immune responses and suggest that NIK dysfunction may lead to weakened inflammatory reactions.

## 1. Introduction

Interleukin 12 (IL-12) is a heterodimeric cytokine mainly produced by macrophages and dendritic cells (DCs) in response to intracellular pathogens and bacterial products. It can also be secreted by adaptive immune cells depending on the immune context. It has powerful effects in the activation and maturation of both cytotoxic CD8 T cells and NK cells [1]. Besides its function in effector immune cells, treatment with IL-12 has been shown to inhibit tumor-induced Treg cell proliferation [2]. Because of its powerful pro-inflammatory features, recent clinical studies have developed strategies for delivering IL-12 safely into the tumor microenvironment, making it a promising tool for tumor immunotherapy [3,4]. While IL-12 plays a crucial role in normal immune responses, its overproduction can lead to the development of autoimmune and chronic inflammatory diseases [5]. Therefore, understanding the mechanisms that regulate IL-12 expression is essential in making advances in the control and treatment of infectious and inflammatory conditions.

The bioactive IL-12 p70 heterodimer is composed of two covalently linked glycosylated chains, p40 and p35, which are encoded by separate genes [6]. The highly coordinated expression of *Il12b* and *Il12a* (which encode the IL-12 p40 and p35 proteins, respectively) to form IL-12 is essential in the initiation of an effective immune response.

IL-12 p40 also forms heterodimers with another protein, p19. The p19/p40 cytokine, IL-23, contributes unique functions in regulating immune responses through the recruitment and activation of inflammatory cells [7]. IL-12 and IL-23 work in concert to regulate cellular immune responses that are critical to host defense and tumor suppression [8]. A critical component in the regulation of these two cytokines is the transcriptional control of the *Il12b* gene.

The *Il12b* promoter contains several transcription factor-binding sites that contribute to gene induction. The DNA-binding elements that have been best characterized bind NF-κB, C/EBP, AP-1, IRF, and NFAT family members [9,10,11,12,13,14]. The most important control elements identified via transient transfection assays in the *Il12b* promoter bind the NF-κB and C/EBP families of transcription factors [11].

In mammals, the NF-κB family consists of five members—c-Rel, p65 (RelA), RelB, NF-κB1 (p50), and NF-κB2 (p52)—that bind to DNA as homodimers and heterodimers [15]. The NF-κB binding site in the *Il12b* gene has been shown to preferentially bind p50 and c-Rel in macrophages [16,17]. In fact, the impaired production of IL-12 in many autoimmune-prone mouse strains has been related to a predominance of the p50/p50 complex binding to the *Il12b* promoter and reduced p50/c-Rel binding [16].

NF-κB transcription factors are present in the cytoplasm in an inactive state, complexed with members of the IκB family. In response to different activators, IκB is phosphorylated by IκB kinases (IKKs), ubiquitinated, and finally degraded by the proteasome. The dual nuclear localization signals of the NF-κB complex are then exposed, and the complex translocates to the nucleus and activates transcription [18]. In addition to this conventional pathway, a noncanonical one involves signaling through NF-κB-inducing kinase (NIK) [19,20]. NIK is a serine/threonine protein kinase expressed in a variety of cell types, such as T cells [21], macrophages [22], B cells, and DCs [23]. This kinase phosphorylates IKKα [24], an event that finally leads to the phosphorylation of p110 and its subsequent processing to p52 [25]. Both pathways (canonical and non-canonical) converge in the specific activation of NF-κB through NIK [16,26,27,28,29], making it a pivotal component in the modulation of NF-κB-mediated signaling.

Besides translocation to the nucleus, accumulating evidence suggests that another level of NF-κB regulation exists, which relies in the activation of the transcriptional activity of NF-κB family members through post-translational modifications [30]. In this regard, we have shown that NIK associates with c-Rel and strongly potentiates its transactivation activity through phosphorylation in T cells [31].

NIK mutant mice, including alymphoplasia (*aly*/*aly*) mice, have proven to be useful tools in unveiling the essential role of NIK in the induction of NF-κB members. The *aly*/*aly* mouse phenotype is caused by an autosomal recessive missense point mutation in the C-terminal domain of NIK [32]. This mutation is located in the position where NIK interacts with IKKα, TNFR-associated factor 2 (TRAF2), TRAF5 [19,33], and c-Rel [31] and has been shown to affect its function in processing p100 [34]. Given its relevant role in the activation of the NF-κB pathway in several cell types, NIK represents an attractive pharmacological target for the treatment of a variety of immune-related diseases. Therefore, defining its role and function within the immune system has become a very important issue.

In this study, we investigated the mechanisms responsible for regulating cytokine synthesis in LPS-stimulated macrophages through NIK, with an emphasis on the induction of *Il12b* transcription. Because most previous studies relied on the over-expression of the signaling components, we attempted, whenever possible, to analyze the properties of endogenous NIK and c-Rel in primary macrophages. For this purpose, we used macrophages isolated from *aly*/*aly* mice and compared them to WT mice.

We found that *aly*/*aly* macrophages are defective in activation, leading to impaired *Il12b* transcription. More importantly, we found that NIK is required for the shuttling of c-Rel to the nucleus and binding to the *Il12b* promoter.

## 2. Materials and Methods

### 2.1. Mice

ALY/NscJcl-aly/aly (*aly*/*aly*) mice were obtained from CLEA Japan, Inc. (Tokyo, Japan), and C57BL/6J mice were obtained from The Jackson Laboratory (Bar Harbor, ME, USA). We used 6–8-week-old female mice in all experiments. All studies used mice according to approved protocols in compliance with the European Communities Council Directive of 24 November 1986 (86/609/EEC) and were approved by the Institutional Animal Care and Use Committee of the CBMSO.

The *aly*/*aly* homozygous mutation was demonstrated by running an ELISA assay to detect serum IgA, since *aly*/*aly* mice lack B220^−^IgA^+^ plasma cells [35]. Microtitration plates (Corning, NY, USA) were coated with goat anti-mouse IgA (Cappel Laboratories, Malvern, PA, USA) and blocked with BSA. After incubation with the serum samples, goat anti-mouse IgA antibody (Zymed Laboratories, San Francisco, CA, USA) was applied. The plates were developed using p-nitrophenyl phosphate (Sigma-Aldrich, St. Louis, MO, USA), and absorbency at 450 nm was measured.

### 2.2. Cells

Peritoneal exudate macrophages were collected 4 days after the i.p. injection of 3% thioglycolate and cultured in endotoxin-free RPMI 1640 (Sigma-Aldrich, St. Louis, MO, USA) supplemented with 2% FBS and 2 mM glutamine. The macrophage cell line RAW 264.7 (American Type Culture Collection, Manassas, VA, USA) was grown in RPMI 1640 supplemented with 5% FBS and 2 mM glutamine. The endotoxin levels in the culture medium were lower than 50 ng/mL, as determined by Limulus amoebocyte lysate assay (Cambrex, East Rutherford, NJ, USA).

### 2.3. Reagents

LPS from *Escherichia coli* serotype O26:B6 and protein A-sheparose were purchased from Sigma-Aldrich (St. Louis, MO, USA), murine rIFN-γ was purchased from Biosource International (Wilmington, NC, USA), and poly I:C was obtained from InvivoGen (San Diego, CA, USA).

The expression plasmids encoding human c-Rel, WT human NIK and the mutant forms NIK-KD (K429A/K430A) and aly NIK, and the empty vector pcDNA3 have been previously described [36]. The reporter plasmid pGL2B-IL-12p40luc containing the sequences from -350 to +55 of the murine IL-12 gene directing the transcription of the firefly luciferase gene has been described previously [11]. The pCMV-β-galactosidase was a kind gift from Charles M. Zacharchuk (Dana-Farber Cancer Institute, Boston, MA, USA). The reporter Gal4-luc contains five tandem repeats of the yeast element Gal4 upstream of the luciferase reporter gene [37]. Gal4-c-Rel 309–588 WT has also been described [38].

### 2.4. Cytokine Determination

The total IL-12 p70, IL-12 p40, IL-23, IL-27, TNF-α, and IL-6 in the supernatants were measured by using Quantikine Mouse ELISA kits from R&D Systems (Minneapolis, MN, USA), following the manufacturer’s instructions.

### 2.5. Flow Cytometry

Samples were resuspended in PBS supplemented with 1% BSA and 1% FCS at a concentration of 10 × 10^6^ cells/mL. Cells were incubated in FcBlock (20 μg/mL) (BD Life Sciences, Franklin lakes, NJ, USA) for 20 min at 4 °C, followed by 30 additional minutes with rat anti-mouse TLR4 (Biolegend, San Diego, CA, USA) or purified rat IgG2a, κ Isotype Control Antibody (Biolegend, San Diego, CA, USA). Alexa 647-goat anti-rat (Invitrogen, ThermoFisher Scientific, Waltham, MA, USA) was used as the secondary antibody. Cells were analyzed using an FACSCalibur (BD Life Sciences, Franklin lakes, NJ, USA), and analysis was performed using FlowJo^TM^ software (Version 9.4.1, BD Life Sciences, Franklin lakes, NJ, USA). Data were obtained on 2 × 10^5^ viable cells.

### 2.6. Real-Time PCR

Total RNA was obtained by using TRIzol reagent (Invitrogen, ThermoFisher Scientific, Waltham, MA, USA), following the manufacturer’s instructions; 1 μg of total RNA was reverse-transcribed into cDNA by the two-step RT kit Gene Amp RNA PCR Core kit (Perkin Elmer, Shelton, CT, USA). Real-time PCR was performed on an ABI Prism 7900HT Sequence Detection System (Applied Biosystems, ThermoFisher Scientific, Waltham, MA, USA) using specific TaqMan MGB probes for murine *Il12a* and *Il12b* (Applied Biosystems, ThermoFisher Scientific, Waltham, MA, USA). mRNA gene expression profiles were normalized according to the Actb mRNA expression of each sample, and the fold increase was calculated using the 2^−∆∆Ct^ method [39].

### 2.7. Transient Transfection and Luciferase Assays

The transcriptional activity in RAW 264.7 cells was measured using reporter gene assays after the transient transfection of exponential growing cells (10^6^ cells/mL in OPTIMEM medium) with the Lipofectamine PLUS reagent (Invitrogen, ThermoFisher Scientific, Waltham, MA, USA) according to the manufacturer’s instructions. We co-transfected 500 ng of pGL2B-IL-12p40luc with 200 ng of pCMV-β-galactosidase and 20 ng of expression vectors (NIK, aly NIK and NIK-KD) or the corresponding empty vector (pcDNA3). In the studies performed to determine the implications of NIK in c-Rel transactivating activity, RAW 264.7 cells were co-transfected with 0.5 μg of Gal4 c-Rel-(309–588) fusion construct, 10 ng of Gal4 luciferase reporter, and 20 ng of WT NIK, aly NIK, or NIK-KD or an identical amount of empty pcDNA3 plasmid. After 4 hr. of incubation, the medium was removed and RPMI 1640 medium containing 5% FBS was added. At 24 hr. after transfection, cells were left untreated or incubated with 1 μg/mL LPS for 6 h. Cells were then harvested and lysed. To measure luminescence, the Dual-luciferase Assay System kit from Promega (Madison, VI, USA) was used, according to the manufacturer’s instructions. β-galactosidase activity was measured using reporter lysis buffer according to the manufacturer’s instructions (Promega, Madison, VI, USA). Data are expressed in relative firefly luciferase units (RLUs) normalized by the relative β-gal units and micrograms of protein.

### 2.8. Western Blot and Immunoprecipitation

We plated 4 × 10^6^ peritoneal macrophages onto 6-well Corning plates. After the indicated treatments (LPS 1 μg/mL or medium alone), cells were washed twice with PBS. In order to obtain whole-cell extracts, 200 μL of lysis buffer (20 mM Tris-HCl, pH 7.8, 150 mM NaCl, 1% Nonidet P-40, 10 mM NaF, 10 mM Na3VO4, and 2 μg/mL of protease inhibitors leupeptin, aprotinin, and pepstatin A) was added and cells were incubated for 10 min in ice.

Nuclear proteins were obtained as described [40]. We separated 15 μg of protein in a 10% SDS–polyacrylamide gel and transferred it to a nitrocellulose membrane (Bio-Rad, Hercules, CA, USA). After blocking with 5% nonfat milk in TBS-T, membranes were washed and incubated overnight with the corresponding antibodies at 4 °C. Antibodies against β-actin, nucleolin, c-Rel, NF-κB p50, NF-κB p65, NIK, phospho-IκBα (Ser32), and IκBα were purchased from Santa Cruz Biotechnology (Santa Cruz, CA, USA). Membranes were then washed and incubated for 60 min with the HRP-linked secondary antibody (ThermoFisher Scientific, Waltham, MA, USA). Bands were visualized with the ECL detection reagent (Amersham Biosciences, Slough, Buckinghamshire, UK).

For the immunoprecipitation of NIK, 2 × 10^6^ RAW 264.7 cells were transfected with 10 μg of pcDNA3-NIK, pcDNA3-alyNIK, pcDNA3-NIK-KD, or empty vector using the jetPEI reagent (Polyplus Sartorius, Illkirch-Graffenstaden, France). Cells were incubated for 48 hr. in RPMI containing 5% FBS. Whole-cell extracts were obtained as explained earlier and immunoprecipitated with anti-NIK (Santa Cruz Biotechnology, Santa Cruz, CA, USA) at 4 °C overnight. Immuno-complexes were recovered with protein A/G PLUS-agarose (Santa Cruz Biotechnology, Santa Cruz, CA, USA) and washed 5 times with lysis buffer. For the detection of NIK and c-Rel, 8% polyacrylamide gels were electrophoretically transferred to nitrocellulose filters (Bio-Rad, Hercules, CA, USA).

The quantification of bands in Western blotting was performed with ImageJ 1.54k software (National Institutes of Health, Bethesda, MD, USA).

### 2.9. Confocal Imaging

Peritoneal macrophages were allowed to adhere to poly-L-lysine-treated chamber polystyrene plates for 1 h (ThermoFisher Scientific, Waltham, MA, USA). Cells were then fixed with PBS–sucrose-formaldehyde (4.4% sucrose, 3.7% formaldehyde in PBS) for 10–20 min, washed with PBS twice, and permeabilized with 0.25% Triton X-100 in distilled water for another 10 min. Following this, cells were treated with 50 mM NH4Cl for 10 min to eliminate autofluorescence. This was followed by blocking with 2% BSA in PBS for 20 min and staining with rabbit anti-c-Rel (Santa Cruz Biotechnology, Santa Cruz, CA, USA) overnight at 4 °C. Cells were washed in PBS and stained with Alexa 488 donkey-anti-rabbit (Invitrogen, ThermoFisher Scientific, Waltham, MA, USA) at room temperature for 1 h. ProLong Gold Antifade Mountant with DNA Stain DAPI (ThermoFisher Scientific, Waltham, MA, USA) was used as the mounting media. Cells that were stained with secondary antibodies alone served as the negative control for unspecific staining. Samples were analyzed using a confocal Zeiss LSM510 inverted microscope (Oberkochen, Germany).

### 2.10. Chromatin Immunoprecipitation Assay

We maintained 6 × 10^6^ cells per point in RPMI with 0.5% FCS for 18 h before stimulation. After treatment with LPS for 1 h, cells were fixed with 1% formaldehyde for 10 min at 37 °C. Cells were lysed in ice-cold lysis buffer (10 mM HEPES, 1.5 mM MgCl_2_, 10 mM KCl, 0.5 mM DTT, 0.1% Nonidet P-40, and protease inhibitor cocktail from Roche) for 10 min. Nuclei pellets were resuspended in nuclear lysis buffer (50 mM Tris-HCl, pH 8, 10 mM EDTA, 1% SDS, and protease inhibitors) and incubated on ice for 10 min. Chromatin was sheared by sonication for 2 rounds of 4 cycles of 30 s ON/30 s OFF with the Bioruptor PLUS at a high power setting (Diagenode, Denville, NJ, USA) and microfuged at 14,000 rpm for 10 min at 8 °C. Extracts were diluted 7 times in dilution buffer (50 mM Tris-HCl, pH 8, 5 mM EDTA, 200 mM NaCl, 0.5% NP-40, and protease inhibitors) and were precleared with salmon-sperm-pretreated protein G agarose (Merck Millipore, Burlington, MA, USA) for 3 h at 4 °C. Precleared lysate was incubated overnight at 4 °C with 3 μg of the corresponding antibody against c-Rel, p65 or p50, or with normal rabbit serum (NRS) as a negative control. NRS, anti-c-RelX, anti-NF-κB p50X, and anti-NF-κB p65X came from Santa Cruz Biotechnology (Santa Cruz, CA, USA).

Immune complexes were collected by adding salmon-sperm-pretreated protein G agarose for 1 h at 4 °C. Washes were performed with wash buffer (20 mM Tris-HCl, pH 8, 2 mM EDTA, 0.1% SDS, 1% NP-40, 500 mM NaCl), and followed by an additional wash in 20 mM Tris-HCl, 2 mM EDTA buffer. For the elution of chromatin complexes, extraction buffer (20 mM Tris-HCl, pH 8, 2 mM EDTA, 2% SDS) was added to samples, and the cross-link reversal took place at 65 °C overnight. Proteins were digested with 150 μg/mL Proteinase K, and DNA was extracted by using the High Pure PCR template preparation kit (Roche, Basel, Switzerland), following the manufacturer’s instructions. PCR amplifications were carried out by using GoTaq polymerase (Promega, Madison, VI, USA). The primers used to detect the consensus region for the NF-κB site in the mouse *Il12b* promoter were as follows: mouse *Il12b* NF-κB forward, 5′-CGTCTATATTCCCTCTGTAT-3′; reverse, 5′-AGTAGAAACTGACTAGTCTC-3′.

### 2.11. Statistical Analysis

Data were analyzed by two factorial univariate or multivariate analyses of variance (ANOVA) with Bonferroni correction. *p* values < 0.05 denote statistical significance.

## 3. Results

### 3.1. Defective Activation of aly/aly Macrophages in Response to TLR Agonists

**Figure 1:**
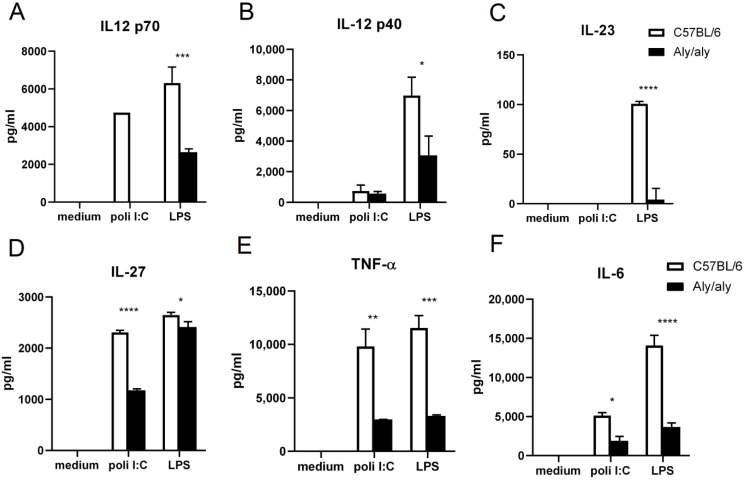
Defective activation of *aly*/*aly* macrophages in response to TLR agonists. Concentrations of IL12 p70 (**A**), IL12p40 (**B**), IL-23 (**C**), IL-27 (**D**), TNF-α (**E**), and IL-6 (**F**) secreted by WT and *aly*/*aly* mouse peritoneal macrophages after in vitro activation for 16 h with 25 μg/mL poli I:C or 1 μg/mL LPS. Values are represented as mean + s.d of three independent experiments each carried out in duplicate. One-way ANOVA and Tukey’s multiple comparison test; * *p* < 0.05, ** *p* < 0.01, *** *p* < 0.001, **** *p* < 0.001.

The activation of macrophages is a critical event that leads to the production of inflammatory mediators. In order to study the involvement of NIK in macrophage activation, some pro-inflammatory stimuli, such as the TLR3 agonist poli I:C and the TLR4 agonist LPS, were used to induce the activation of peritoneal macrophages from WT and *aly*/*aly* mice. As shown in Figure 1, the production of IL-12 p70, IL-12 p40, IL-23, IL-27, TNF-α, and IL-6 is impaired in *aly*/*aly* macrophages stimulated with LPS. In response to poli I:C, IL-12 p70, IL-27, TNF-α, and IL-6 secretion is dampened in *aly*/*aly* cells. Genes that encode for IL-27, TNF- α, and IL-6 are known to be regulated by NF-κB transcription factors [41,42,43]. IL-12 p70 and IL-23 share the p40 subunit, the expression of which is tightly regulated by NF-κB family members [11].

### 3.2. The aly/aly Mutation Affects the Transcription of the Il12b and Il12a Genes

**Figure 2:**
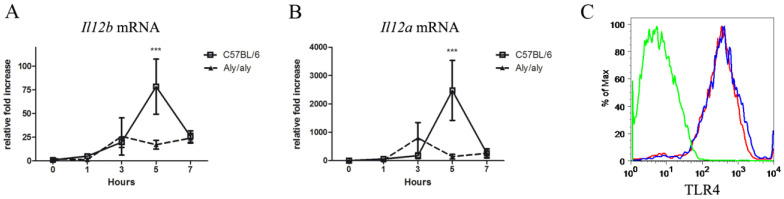
The *aly*/*aly* mutation affects the transcription of the *Il12b* and *Il12a* genes. *Il12b* (**A**) and *Il12a* (**B**) mRNA levels in peritoneal macrophages from WT and *aly*/*aly* mice in response to 1 μg/mL LPS as determined by quantitative real-time PCR. Values are represented as mean fold increase ± s.d. A representative assay from three independent experiments is shown. Two-way ANOVA and Bonferroni post hoc tests; *** *p* < 0.001. (**C**) Histogram plot of F4/80^+^ mouse peritoneal macrophages showing the expression of TLR4. A representative analysis from three independent experiments is shown. Red line, *aly*/*aly* macrophages; blue line, WT macrophages; green line, isotype control antibody.

The fact that the secretion of all these cytokines by activated macrophages was affected by the presence of a point mutation in NIK (*aly*/*aly*) led us to investigate in greater detail the transcriptional events that take place during macrophage activation and culminate in NF-κB-dependent gene expression. Because IL-12 p70 consists of p40 and p35 subunits in a 1:1 molar ratio, our data likely suggest that NIK regulates Il12b and Il12a gene expression. Therefore, we analyzed the kinetic expression pattern of Il12b and Il12a mRNA in LPS–treated macrophages from WT and *aly*/*aly* mice. As shown in Figure 2A,B, respectively, the transcription of both genes is impaired in *aly*/*aly* macrophages 5 h after LPS stimulation.

Since LPS signals through its receptor TLR4, we next proved that the differential expression of Il12b and Il12a mRNA in *aly*/*aly* macrophages cannot be attributable to a lower expression of surface TLR4. As shown in Figure 2C, the surface levels of TLR4 in *aly*/*aly* macrophages were comparable to WT cells.

### 3.3. NIK Regulates Il12b Gene Transcription in Macrophages Through the Stimulation of c-Rel Transactivating Activity

**Figure 3:**
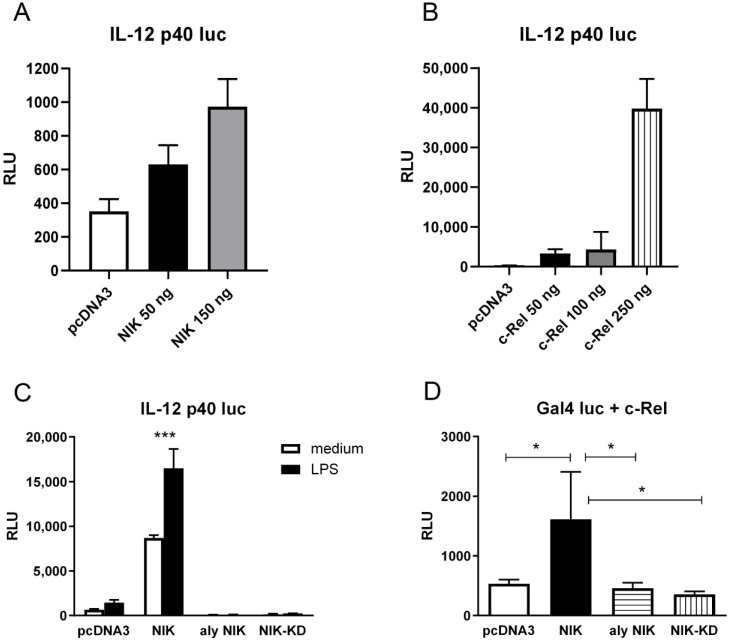
NIK is necessary for the transcription of the *Il12b* gene in macrophages. The mechanism involves the stimulation of c-Rel transactivating activity. (**A**) RAW 264.7 cells were transiently transfected with the IL-12p40luc reporter gene together with increasing amounts of wild-type NIK or empty pcDNA3 plasmid. Transfection efficiency was evaluated with the pCMV-β-galactosidase plasmid. This Figure shows the pooled data of two independent experiments. (**B**) RAW 264.7 cells were transiently transfected with the IL-12p40luc reporter gene together with increasing amounts of c-Rel plasmid. The results shown are the pooled data of two independent experiments. (**C**) RAW 264.7 cells were transiently transfected with the IL-12p40luc reporter gene together with wild-type NIK, aly NIK, NIK-KD, or an identical amount of empty pcDNA3 plasmid. Cells were left untreated or stimulated with 1 μg/mL LPS for 6 h. The results shown are representative of four experiments performed. Two-way ANOVA and Bonferroni post hoc tests; *** *p* < 0.001 (*n* = 2). (**D**) RAW 264.7 cells were co-transfected with Gal4 c-Rel-(309–588) fusion construct, Gal4 luciferase reporter and WT NIK, aly NIK, NIK-KD, or an identical amount of empty pcDNA3 plasmid. The results are representative of two experiments performed. Data are expressed in RLUs normalized by the relative β-gal units and by μg of protein. Values are represented as mean RLU + s.d. One-way ANOVA and Tukey’s multiple comparison test; * *p* < 0.05 (*n* = 2).

To gain further understanding of the mechanism behind the LPS-mediated induction of *Il12b* in macrophages, we used a well-established transient transfection model in the murine myeloid cell line RAW 264.7. Cells were transfected with a reporter construction that contains the luciferase gene under the control of the murine *Il12b* promoter. In the first set of experiments, we over-expressed either NIK or c-Rel by co-transfection with the *Il12b* promoter reporter. As seen in Figure 3A,B, the over-expression of both NIK and c-Rel strongly stimulates *Il12b* transcription in a concentration-dependent manner.

Next, RAW 264.7 cells were co-transfected with expression plasmids that encode WT NIK, *aly*/*aly* NIK, and a mutant form of NIK called NIK-KD, which lacks kinase activity, and cells either were left untreated or were treated with LPS. Figure 3C shows that the *Il12b* promoter was responsive to LPS stimulation (around 2-fold), but this response was greatly enhanced when WT-NIK was over-expressed, whereas the over-expression of the mutant forms *aly*/*aly* NIK and NIK-KD inhibited *Il12b* transcription. Again, this illustrates that NIK expression is required for *Il12b* transcription.

To further explore the molecular mechanism by which NIK regulated *Il12b* transcription, we analyzed c-Rel transactivating activity. We had previously shown that NIK phosphorylated c-Rel in T cells and potentiated its transactivating function [31], but we wanted to test whether this regulation occurred in macrophages as well. Therefore, we transfected either WT-NIK, *aly*/*aly* NIK, or NIK-KD together with Gal4-c-Rel along with a Gal4-luc reporter plasmid, and luciferase activity was recorded. As shown in Figure 3D, the over-expression of NIK up-regulated c-Rel transactivating activity in RAW 264.7 cells. As expected, the over-expression of NIK-KD did not have any effect on c-Rel transactivating activity, since this mutant form lacks kinase activity. However, no effect was seen when the *aly*/*aly* NIK form was over-expressed, even though this mutant form possesses an intact kinase domain.

### 3.4. Aly/aly NIK Impedes Complete NF-κB Activation and Prevents Binding of NIK to c-Rel After LPS Challenge

**Figure 4:**
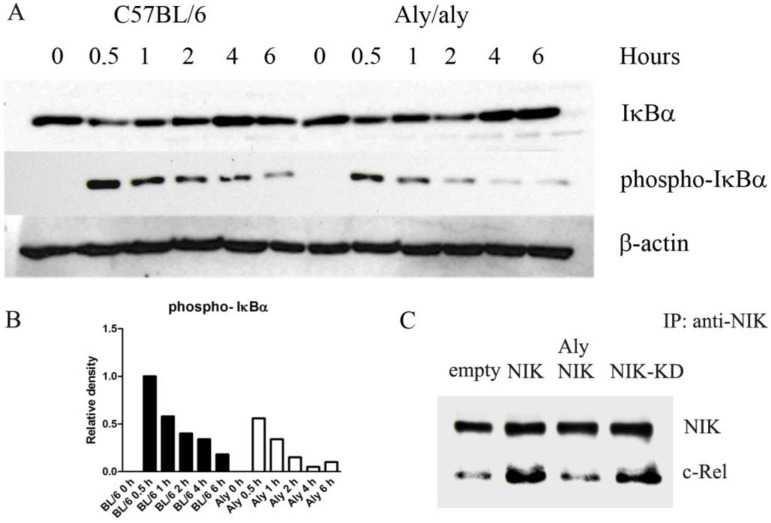
The *aly*/*aly* mutation impedes complete IκBα activation after LPS challenge and prevents the binding of NIK to c-Rel. (**A**) Mouse peritoneal macrophages from WT and *aly*/*aly* mice were exposed to 1 μg/mL LPS. Protein extracts were obtained at distinct time points and analyzed by Western blot to detect the phosphorylation of IκBα. Blotting is representative of three independent experiments (Supplementary Materials). (**B**) The quantification of Western blot bands for phospho-IκBα. The relative density was normalized with the corresponding IκBα and β-actin bands. (**C**) RAW 264.7 cells were transfected with pcDNA3-NIK, pcDNA3-alyNIK, pcDNA3-NIK-KD, or empty vector. Then, 48 h later, lysates were immunoprecipitated with anti-NIK serum and fractionated by SDS-PAGE. The presence of NIK and c-Rel in the immunocomplexes was then analyzed by immunoblotting. Blotting is representative of three independent experiments (Supplementary Materials).

The signal-induced phosphorylation of IκBα is a key event that triggers the cascade that culminates in NF-κB activation, and NIK has been shown to act upstream of IκBα, activating IKKα [21]. Therefore, we checked whether the *aly*/*aly* mutation affected IκBα phosphorylation under LPS challenge. As shown in Figure 4A,B, the phosphorylation of IκBα was not as efficient in *aly*/*aly* macrophages as it was in WT controls.

A second level of regulation of NF-κB activation by NIK relies on binding to the transcription factors and enhancing their transactivation activity. We have shown that NIK interacts with c-Rel in the region 771-949 of NIK [31], and this interaction domain maps precisely in the area where the *aly*/*aly* mutation is located. We hypothesized that the *aly*/*aly* mutation impedes the binding of c-Rel to NIK. To test this, RAW 264.7 cells were transfected with either WT-NIK, *aly*/*aly* NIK, NIK-KD, or empty vector, and lysates were immunoprecipitated with anti-NIK. As expected, the over-expression of NIK allows the pulling down of a higher amount of c-Rel than the amount obtained by immunoprecipitating endogenous NIK (Figure 4C). The same was observed when NIK-KD was over-expressed (Figure 4C). This result was expected, since mutations in the kinase domain of NIK should not interfere with the binding of c-Rel. However, the over-expression of the *aly*/*aly* form of NIK does not reflect any differences with respect to the amount of c-Rel pulled down by endogenous NIK (Figure 4C), showing that the *aly*/*aly* mutation prevents the binding of c-Rel to NIK.

### 3.5. Accumulation of c-Rel in the Nucleus upon LPS Challenge Is Inhibited in aly/aly Macrophages

Since the binding of c-Rel and NIK is impaired in *aly*/*aly* mutants, we next checked whether this mutation also affected the dynamics of translocation to the nucleus of NIK and NF-κB members. NIK has been shown to shuttle constitutively between the cytoplasm and the nucleus [44], a process that depends on the presence of functional nuclear localization and export signals [45]. Any change in this dynamic process would potentially interfere with the translocation of transcription factors that bind to NIK.

**Figure 5:**
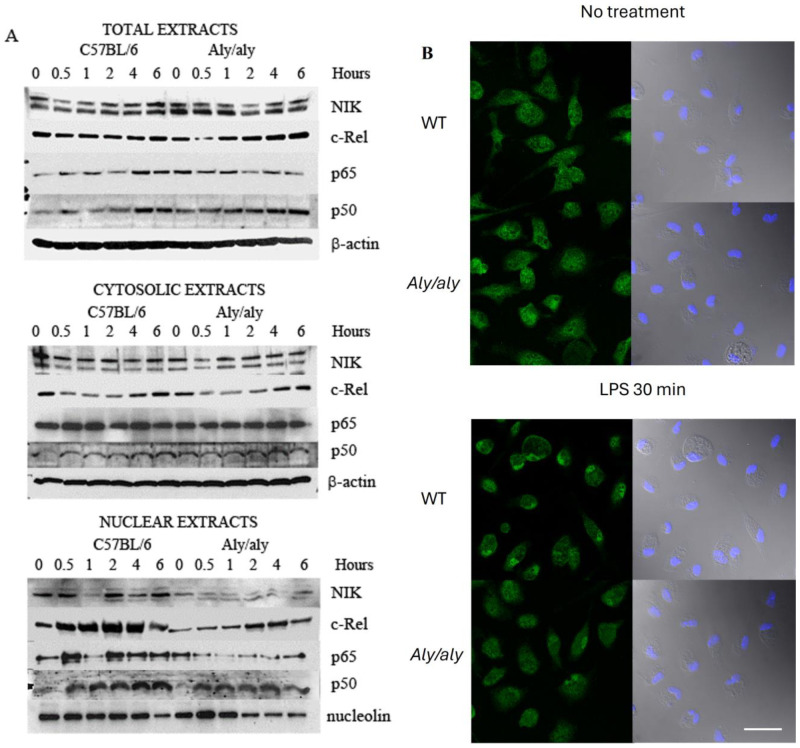
The accumulation of c-Rel in the nucleus upon LPS challenge is inhibited in *aly*/*aly* macrophages. (**A**) Mouse peritoneal macrophages from WT and *aly*/*aly* mice were exposed to 1 μg/mL LPS for 6 h. Total, cytoplasmic, and nuclear protein extracts were obtained at distinct time points and analyzed by Western blotting to detect NIK, c-Rel, p65, and p50. β-actin was used as the loading control in total and cytoplasmic extracts. Nucleolin was used to normalize for protein concentration in nuclear extracts. Blots are representative of three independent experiments (Supplementary Materials). (**B**) Mouse peritoneal macrophages from WT and *aly*/*aly* mice were left untreated or were treated with LPS for 30 min. Cells were then fixed and stained for c-Rel (left panels). DAPI/phase contrast is shown on the right panels. Representative sections of confocal stacks show the accumulation of c-Rel in the nucleus after LPS challenge. Bar: 15 μm.

Immunoblot analysis revealed that NIK appears as a doublet (Figure 5A), possibly as a result of autophosphorylation and/or phosphorylation by activated IKK-α. Nuclear and cytoplasmic Western blotting verified that NIK is present both in the cytoplasm and nucleus of WT macrophages. Moreover, WT-NIK disappears from the nucleus upon stimulation and then returns to normal levels. The amount of nuclear NIK was found to be considerably reduced in *aly*/*aly* cells despite showing normal levels of NIK in total protein extracts (Figure 5A).

As expected, c-Rel was present in the cytoplasm of WT macrophages and translocated to the nucleus after LPS activation. This was shown by a great increase in nuclear c-Rel, peaking at 2 hr., with a concomitant decrease in its cytoplasmic levels (Figure 5A). The total amount of c-Rel was similar in *aly*/*aly* macrophages, but interestingly, when comparing *aly*/*aly* to WT macrophages, we observed a delay in the translocation of c-Rel to the nucleus in *aly*/*aly* cells (Figure 5A). This is in line with the fluorescence microscopy data that showed the accumulation of c-Rel in the nucleus of WT macrophages 30 min after LPS challenge, while little or no accumulation was found in the nucleus of *aly*/*aly* cells (Figure 5B).

With respect to the other NF-κB family members, we found the accumulation of p65 in the nucleus of WT macrophages after LPS stimulation, and this translocation was also severely impaired in *aly*/*aly* cells (Figure 5A). When looking at p50, no changes in protein expression or the dynamics of translocation were evident between the two strains (Figure 5A).

### 3.6. c-Rel Is Bound to the NF-κB Consensus Sequence in the Il12b Promoter After LPS Challenge in Mouse Peritoneal Macrophages and the aly/aly Mutation Results in p65 Being Recruited Instead

In order to determine which NF-κB complexes were being recruited to the NF-κB consensus sequence in the *Il12b* promoter in vivo, we performed a ChIP assay in peritoneal macrophages from *aly*/*aly* and WT mice in response to LPS. The NF-κB site located at position −117 to −107 of the murine *Il12b* promoter has been characterized as being responsible for *Il12b* gene induction upon LPS stimulation in mouse macrophages [9]. Therefore, we designed primers that amplified the region from position −135 to −65.

**Figure 6:**
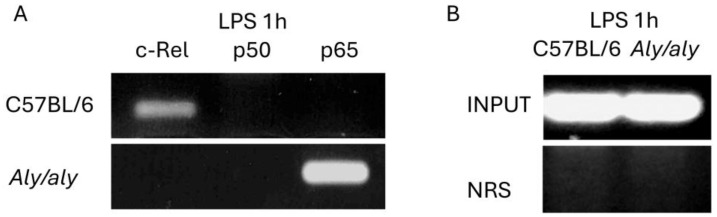
c-Rel is recruited to the *Il12b* promoter in vivo after LPS challenge in mouse peritoneal macrophages. The *aly*/*aly* mutation results in p65 being recruited instead. (**A**) Cells were treated with LPS (1 μg/mL) for 1 h, and after cross-linking with formaldehyde for 30 min, a ChIP assay was performed (see Materials and Methods for details). (**B**) Input, 1% of total chromatin used to verify equal loading of chromatin components before precipitation. Immunoprecipitation performed with Normal Rabbit Serum (NRS) was used to serve as negative controls. Three independent experiments were performed, with similar results. The results from one experiment are shown (Supplementary Materials).

As shown in Figure 6, 1 h after LPS challenge, c-Rel was bound to the NF-κB consensus sequence in the *Il12b* promoter in WT macrophages, whereas the transcription factor bound to the site in *aly*/*aly* cells was p65. Immunoprecipitation with nonspecific NRS did not lead to the recruitment of any transcription factor to the *Il12b* promoter.

## 4. Discussion

We have shown here that NIK plays a very important role in cytokine secretion in macrophages activated by TLRs. We found that the induction of several pro-inflammatory cytokines was partially or almost completely reduced in macrophages harboring the *aly*/*aly* mutation (Figure 1). All these genes are regulated to some extent by NF-kB family members [11,41,42,43]. The fact that p65 and p50 binding to the DNA in *aly*/*aly* mice has been shown to be normal [46] led us to consider the role of other potential members of the family. C-Rel seemed like a good candidate, since it is involved in the transcriptional regulation of the IL-27 *p28* gene in macrophages [41] and is selectively required for *Il12b* induction in LPS-stimulated macrophages [17].

Even though our results differ from those of Smith et al. [47], who reported that NIK was not required for LPS-induced cytokine expression in human macrophages, the apparent discrepancy can be easily explained when looking in greater detail at the methodology employed by these authors. Smith et al. infected primary human M-CSF-differentiated macrophages with Adenovirus-NIK-KD before stimulation with LPS. This molecular approach serves to over-express the kinase-deficient form of NIK but does not eliminate endogenous NIK, which could potentially phosphorylate and/or bind to c-Rel. Moreover, NIK-KD can still bind c-Rel, whereas *aly* NIK cannot [31]. Our study makes use of primary cells from *aly*/*aly* mice whenever possible in order to avoid artifacts due to the over-expression of proteins.

We have demonstrated that *Il12b* gene transcription is impaired in *aly*/*aly* macrophages after exposure to the TLR4 agonist LPS. Consistent with the results of Boddupalli et al. [48], the over-expression of c-Rel in RAW 264.7 cells stimulated *Il12b* transcription (Figure 3B). The over-expression of NIK had the same effect (Figure 3A), but the mutant forms NIK-KD and *aly*/*aly* NIK failed to induce *Il12b* transcription (Figure 3C). In addition, the over-expression of NIK up-regulated c-Rel transactivating activity (Figure 3D). Despite the limitations of protein over-expression, we consider it a very useful tool to observe the effects of NIK in c-Rel activation in a homogeneous cell population. It has provided us with insights into its molecular mechanisms and potential impact on cellular processes. These experiments pointed us in the right direction when looking for a molecular mechanism that served to explain the relevance of the interaction between NIK and c-Rel in the activation of *Il12b* in macrophages.

We then hypothesized that the *aly*/*aly* mutation could affect *Il12b* transcription at various levels: by interfering with the signaling pathway that leads to NF-κB activation, and more specifically, by preventing the binding of c-Rel to NIK and therefore impeding its phosphorylation.

The identification of NIK as a TRAF and IKKα-interacting kinase suggested that this enzyme was central to the NF-κB activation pathway [33,36]. This view was confirmed by many subsequent studies that associated NIK with NF-κB activation by multiple and diverse stimuli. The interaction between NIK and IKKα results in the phosphorylation of IKKα, which, in turn, phosphorylates IκBα at Ser32 [24]. This is an essential event for the release of active NF-κB. IκBα degradation and subsequent NF-κB-DNA binding activity has been shown to be deficient in *aly*/*aly* MEFs [49,50]. In concordance with these results, we have found lower levels of phospho-IκBα in *aly*/*aly* macrophages exposed to LPS (Figure 4A,B), an observation that points directly to defective IKKα activation.

But, more importantly, the *aly*/*aly* mutation is located in the position where NIK interacts with IKKα [33] and c-Rel [31], and an important level of regulation of *Il12b* transcription depends on c-Rel transactivation by NIK [31]. Thus, it is possible that the interaction of NIK with IKKα may serve to recruit NIK into the macromolecular environment of the signalosome, where NIK may interact with c-Rel, contributing to its transactivation. Our immunoprecipitation studies demonstrated that the binding of NIK to c-Rel was not efficient enough when the *aly*/*aly* form of NIK was over-expressed (Figure 4C), therefore providing a molecular mechanism to explain the dysregulation of *Il12b* gene transcription in *aly*/*aly* macrophages. The defective interaction between *aly* NIK and IKKα in *aly*/*aly* macrophages, together with the defective binding of NIK and c-Rel, would ultimately result in lower c-Rel transactivation, as we have observed in our transfection experiments.

More importantly, we believe that NIK plays a role independently of its kinase activity, as has been proven in other studies [51,52]. The shuttling of both NIK and c-Rel to the nucleus was shown to be impaired in *aly*/*aly* macrophages in response to LPS (Figure 5), resulting in a lesser amount of c-Rel being available for binding to the *Il12b* promoter in *aly*/*aly* cells. This was further confirmed by ChIP assays (Figure 6). NIK has been shown to shuttle constitutively between the cytoplasm and the nucleus [44], and the nucleocytoplasmic distribution of NIK has already been shown to depend on both nuclear import and export signal domains [45]. The presence of the *aly*/*aly* mutation could be interfering, by an unknown mechanism, with NIK shuttling, resulting in a lesser amount of NIK being accumulated in the nucleus of *aly*/*aly* macrophages, thus recruiting less c-Rel with it.

Another mechanism is required to explain the lower levels of c-Rel encountered in the nucleus of *aly*/*aly* macrophages after LPS exposure. The intra-nuclear degradation of NF-κB proteins has already been proposed as a mechanism to regulate the NF-κB-dependent transcription in macrophages [53]. Since the binding of NIK and c-Rel is not as efficient in *aly*/*aly* macrophages as it is in WT cells, unbound nuclear c-Rel could be easily accessible for degradation in *aly*/*aly* cells, resulting in lesser amounts of c-Rel in the nucleus. On the other hand, we cannot rule out the possibility of a more efficient c-Rel exit to the cytoplasm in *aly*/*aly* macrophages.

On the other hand, the *aly*/*aly* mutation has been demonstrated to impair NIK’s capacity to interact with p100, leading to a failure in processing p100 into p52 [34,50]. Consequently, the accumulated p100 may act as an inhibitor by binding to other subunits and sequestering them in the cytoplasm, thereby diminishing their DNA-binding activity.

When looking more specifically at the transcriptional regulation of the *Il12b* promoter, we found that c-Rel was bound to the NF-κB consensus sequence in WT mice after 1h exposure to LPS, whereas in *aly*/*aly* macrophages, the main transcription factor bound to the promoter was p65 (Figure 6). The NF-κB–binding sites within the *Il12b* promoter exhibit similar binding affinities for both p65/p50 and c-Rel/p50 heterodimeric complexes [17]. However, c-Rel alone has been identified as critical to *Il12b* production in macrophages activated via various TLRs or infected with intracellular pathogens. These observations highlight c-Rel as a key and specific regulator of *Il12b* gene expression, setting it apart from the more ubiquitously acting p65 [17]. Considering this, our Western blot and chromatin immunoprecipitation assays explain the defective transcription of *Il12b* in *aly*/*aly* cells: lesser amounts of c-Rel in the nucleus of *aly*/*aly* macrophages may finally result in the lesser binding of c-Rel to the *Il12b* promoter and in the recruitment of p65 instead. The binding of p65 to DNA is not impaired in *aly*/*aly* macrophages [46]. Therefore, in the absence of c-Rel, despite the low amount of nuclear p65 in *aly*/*aly* macrophages, this transcription factor might be capable of binding to the NF-κB consensus sequence in the *Il12b* promoter. Our results demonstrate that p65 is dispensable for *Il12b* transcription, as previously suggested [16,17].

It is important to note that although *Il12b* transcription depends strongly on c-Rel in LPS-stimulated macrophages [17], it is not c-Rel-dependent in all cell types. For instance, the induced expression of IL-12 p70 in LPS-stimulated DCs depends on the transcriptional regulation of *Il12a* rather than *Il12b* [54]. However, the induced expression of *Il12a* in DCs is regulated directly by c-Rel complexes binding to the *Il12a* promoter [54]. Therefore, it can be hypothesized that NIK plays a role in the activation of *Il12a* in DCs by means of its interaction with c-Rel.

The NF-κB family is a critical mediator of inflammatory responses in both mouse and human macrophages. All studies reported here were performed in mouse cells and should be carefully considered before being escalated to humans. However, the regulation of the *IL12B* gene is likely to be similar between mouse and human cells, since the structure of the promoter is quite homologous between species [55] and the NF-κB-like element is highly conserved.

## 5. Conclusions

This study provides a molecular basis for the selective regulation of *Il12b* gene transcription by c-Rel and NIK in macrophages in response to LPS. It provides a mechanistic explanation for the defective IL-12 production in *aly*/*aly* macrophages. Our findings suggest that a complete functional NIK is required for the transcription of *Il12* genes. NIK is required for c-Rel nuclear translocation and the binding of this transcription factor to the NF-κB consensus sequence in the *Il12b* promoter upon LPS challenge.

Our findings reveal important new details about the regulation of the innate immune response in macrophages, uncovering a previously unrecognized NIK–c-Rel signaling pathway that plays a key role in regulating IL-12 expression. Considering the critical role of IL-12 in regulating Th1 immunity and the risk of dysregulated IL-12/Th1 responses in inflammatory diseases, identifying NIK as a key regulator of c-Rel–dependent IL-12 production has significant implications for host defense and the management of inflammatory conditions. As IL-12 is a major target for clinical therapies in inflammatory diseases, our findings introduce a novel target that could offer a precise approach to modulating IL-12 responses effectively.

## Data Availability

The data used to support the findings of this study are available from the corresponding author upon request.

## Supplementary Materials

The following supporting information can be downloaded at: https://www.mdpi.com/article/10.3390/biology14010033/s1, Figure S1: Western blotting from Figure 4A and Figure 5A.
