# Supplementary material for: NF-κB-Inducing Kinase Is Essential for Effective c-Rel Transactivation and Binding to the Il12b Promoter in Macrophages
Source: Biology (Basel). 2025 Jan 3;14(1):33. doi: 10.3390/biology14010033 (PMC11760456; doi:10.3390/biology14010033)

Fig 4 A, lane 1. IκBα

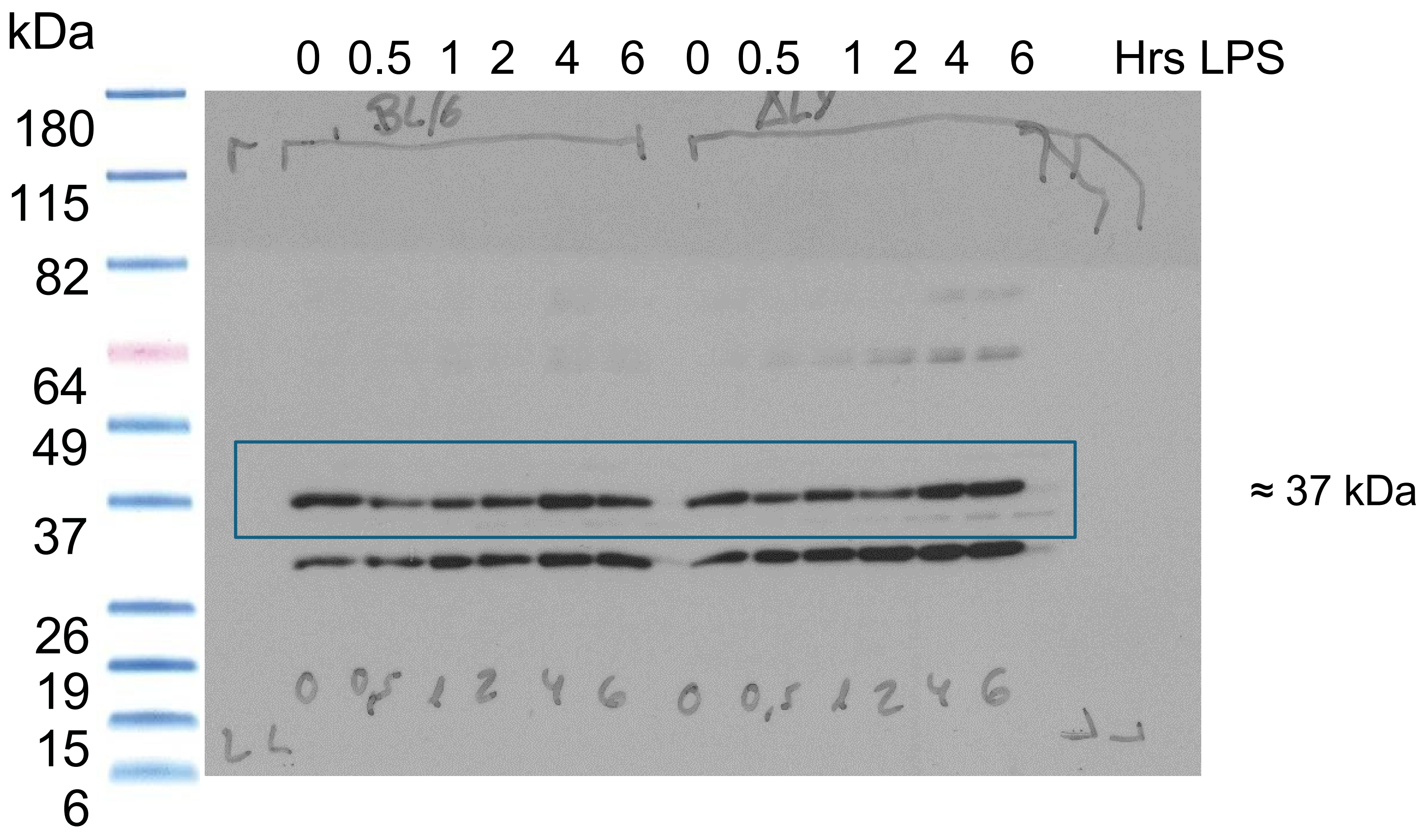

Fig 4 A, lane 2. PhosphoIκBα

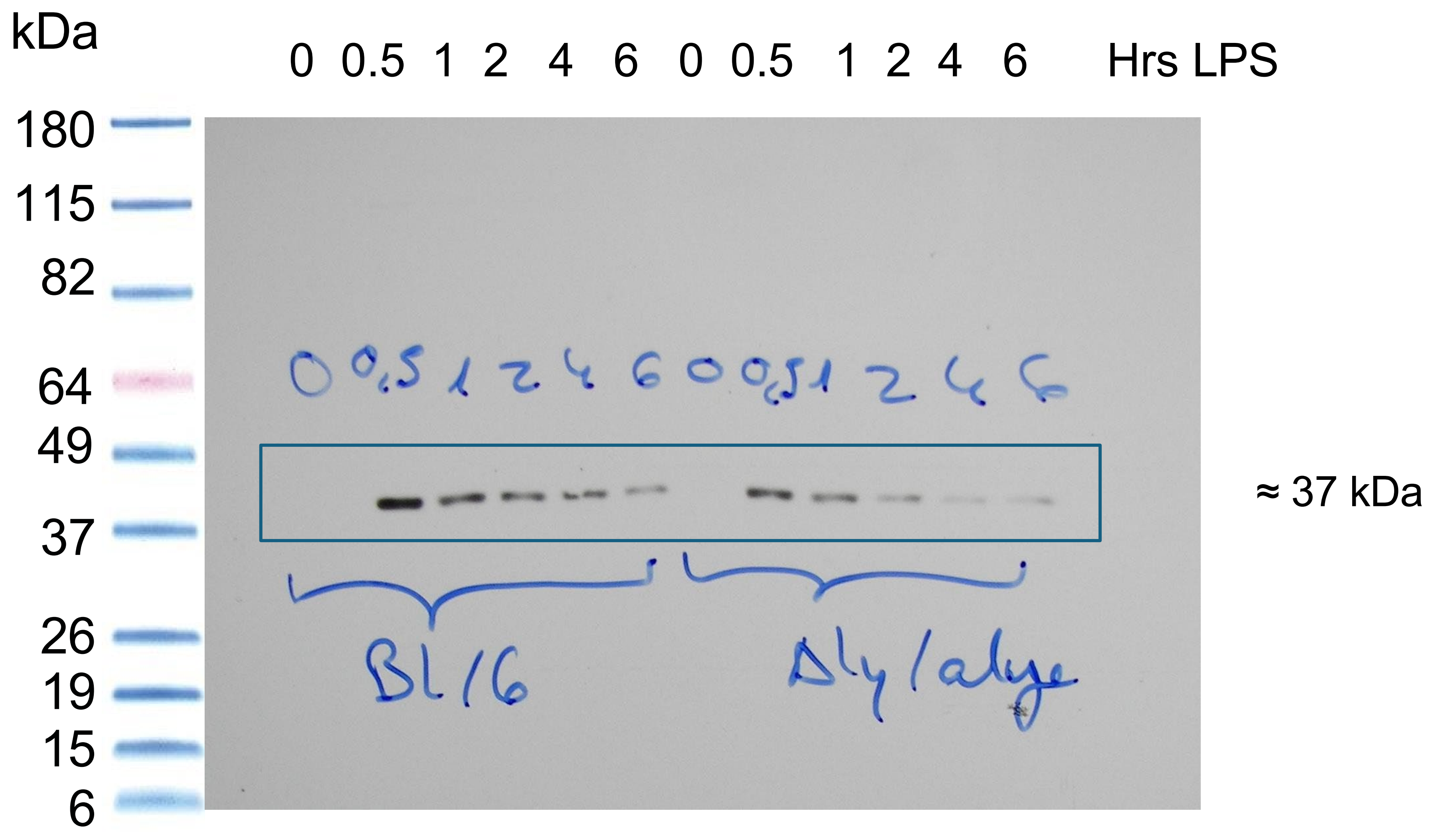

Fig 4 A, lane 3. β-actin

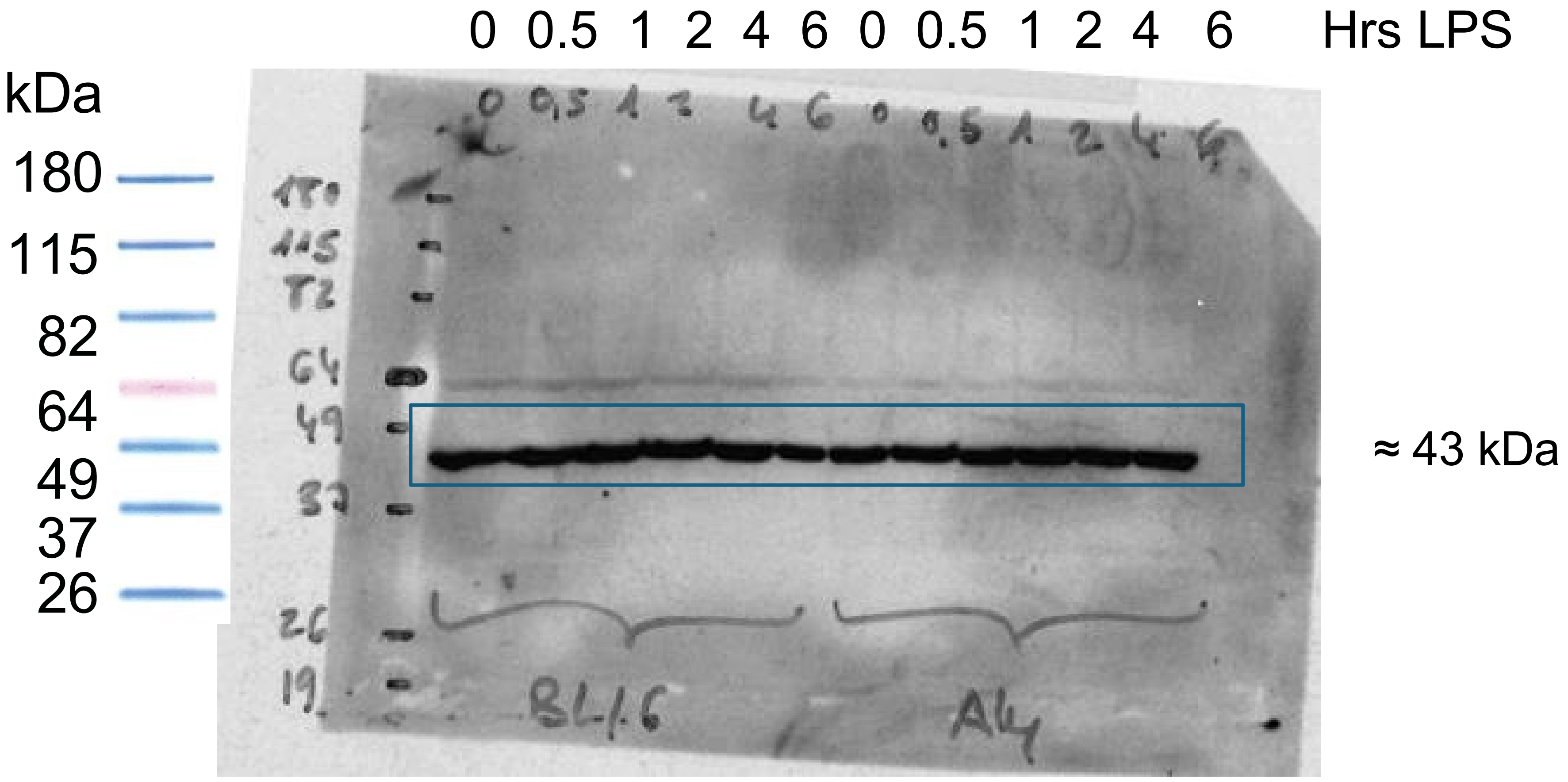

Fig 4 C, lane 1. NIK

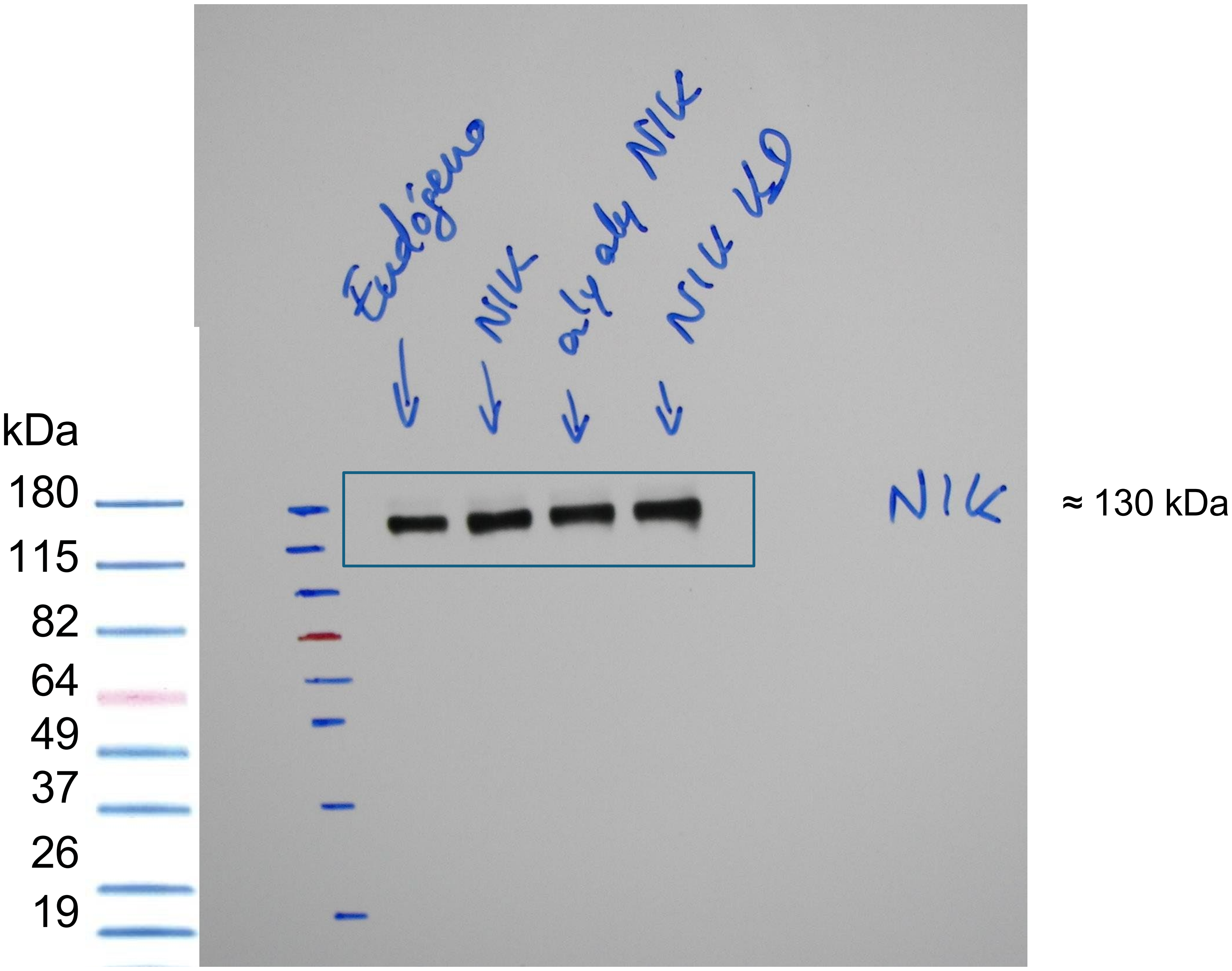

Fig 4 C, lane 2. c-Rel

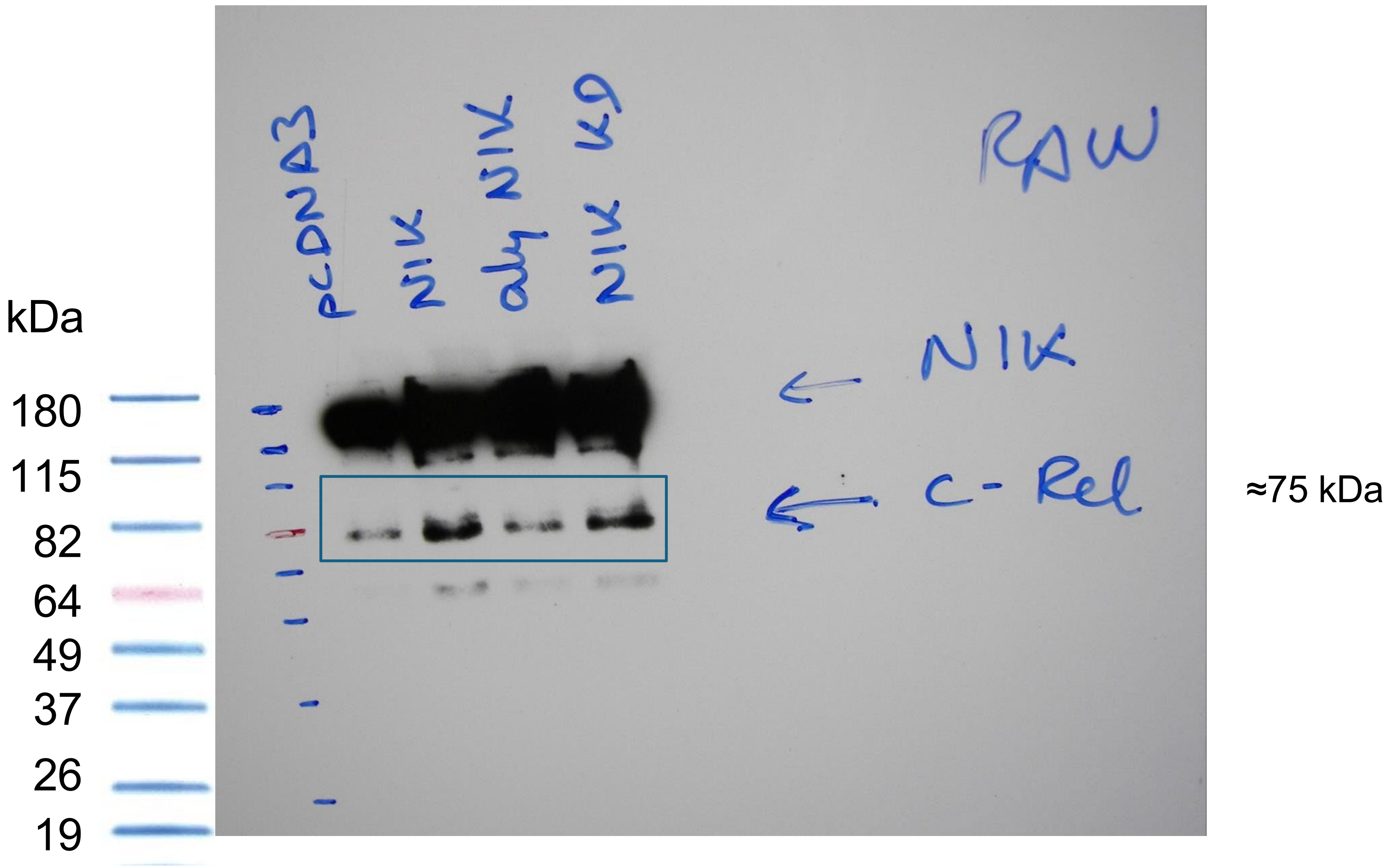

Fig 5 A, lane 1. Total extracts. NIK

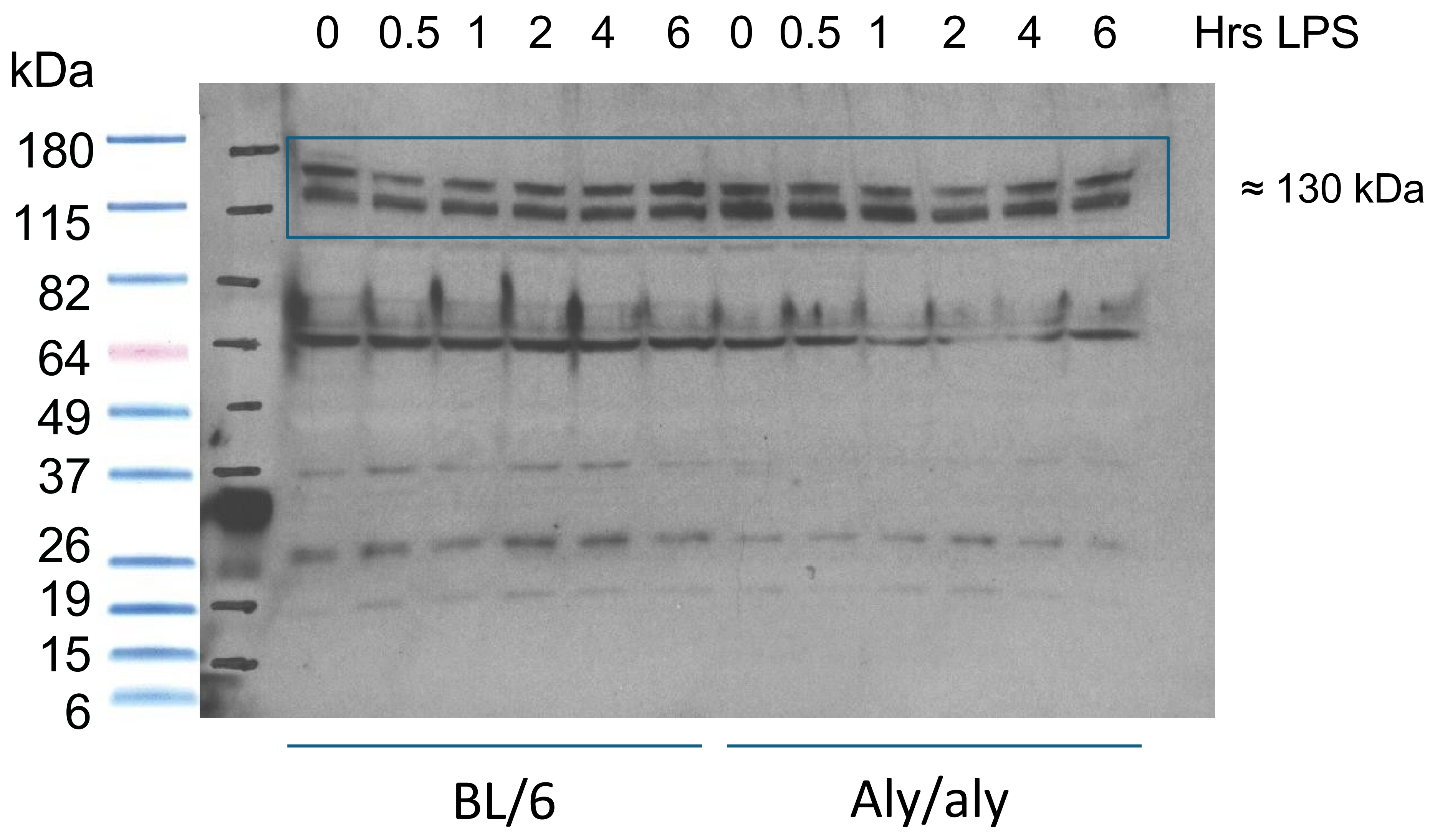

Fig 5 A, lane 2. Total extracts. c-Rel

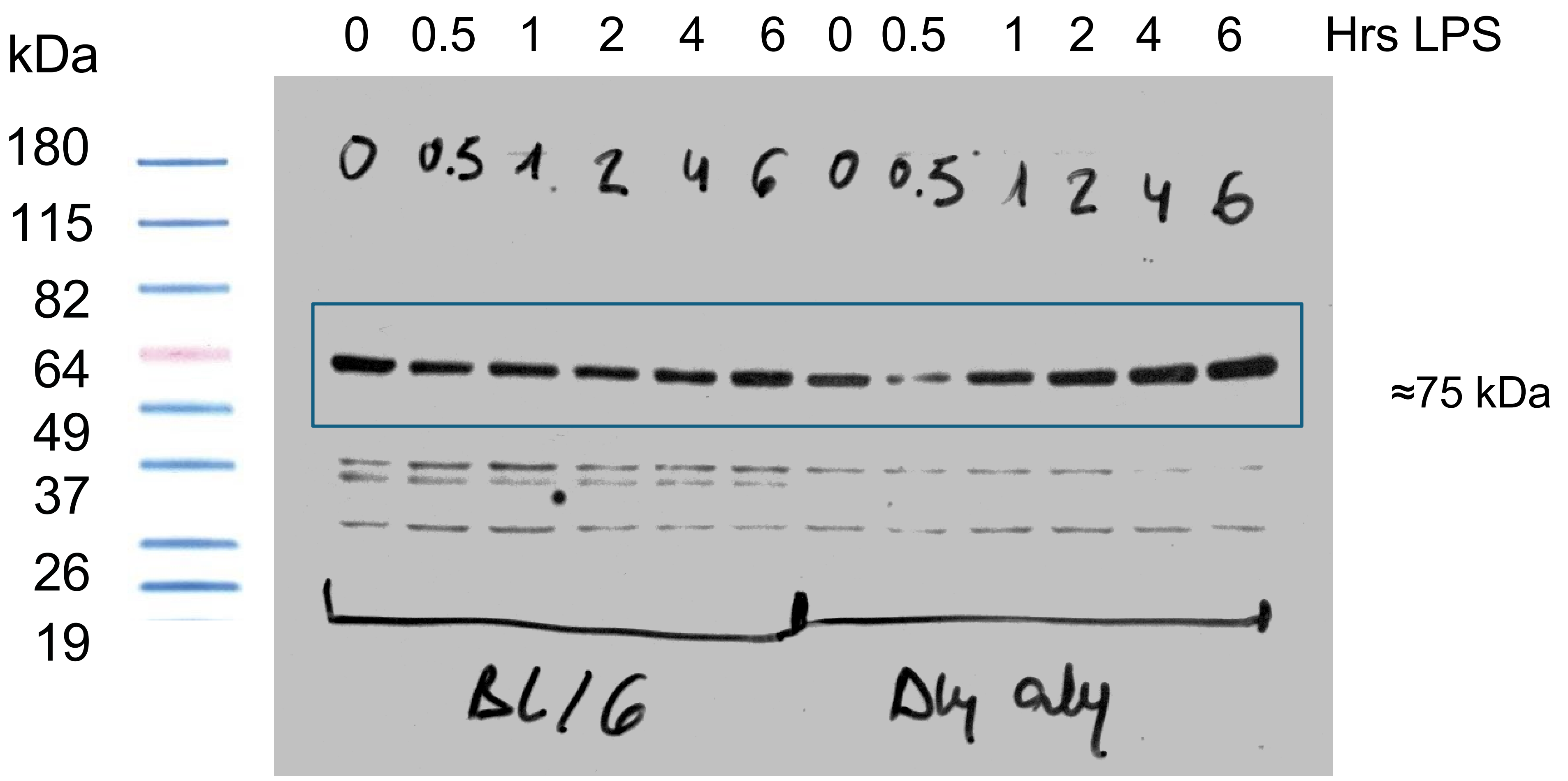

Fig 5 A, lane 3. Total extracts. p65

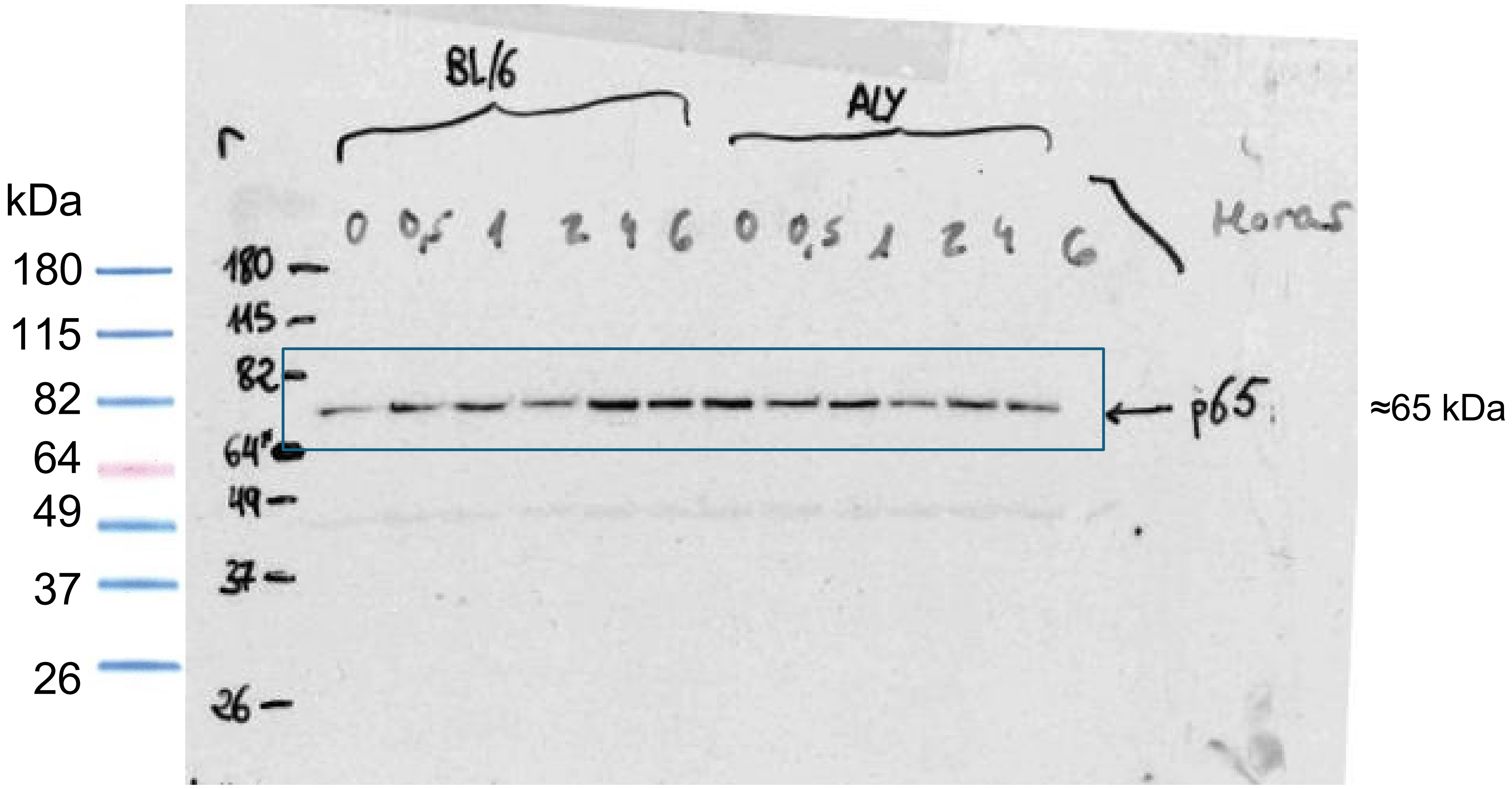

Fig 5 A, lane 4. Total extracts. p50

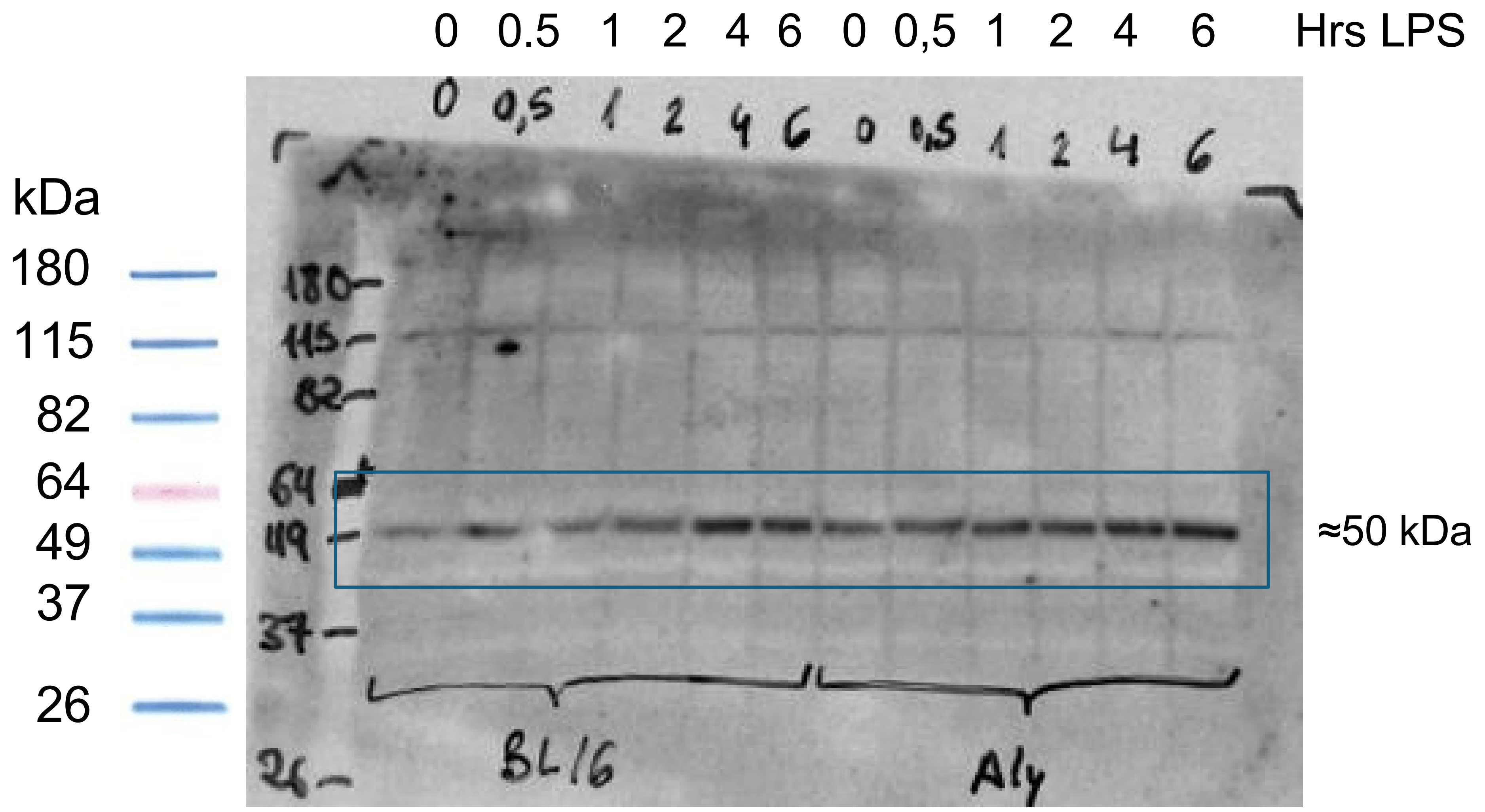

Fig 5 A, lane 5. Total extracts. β-actin

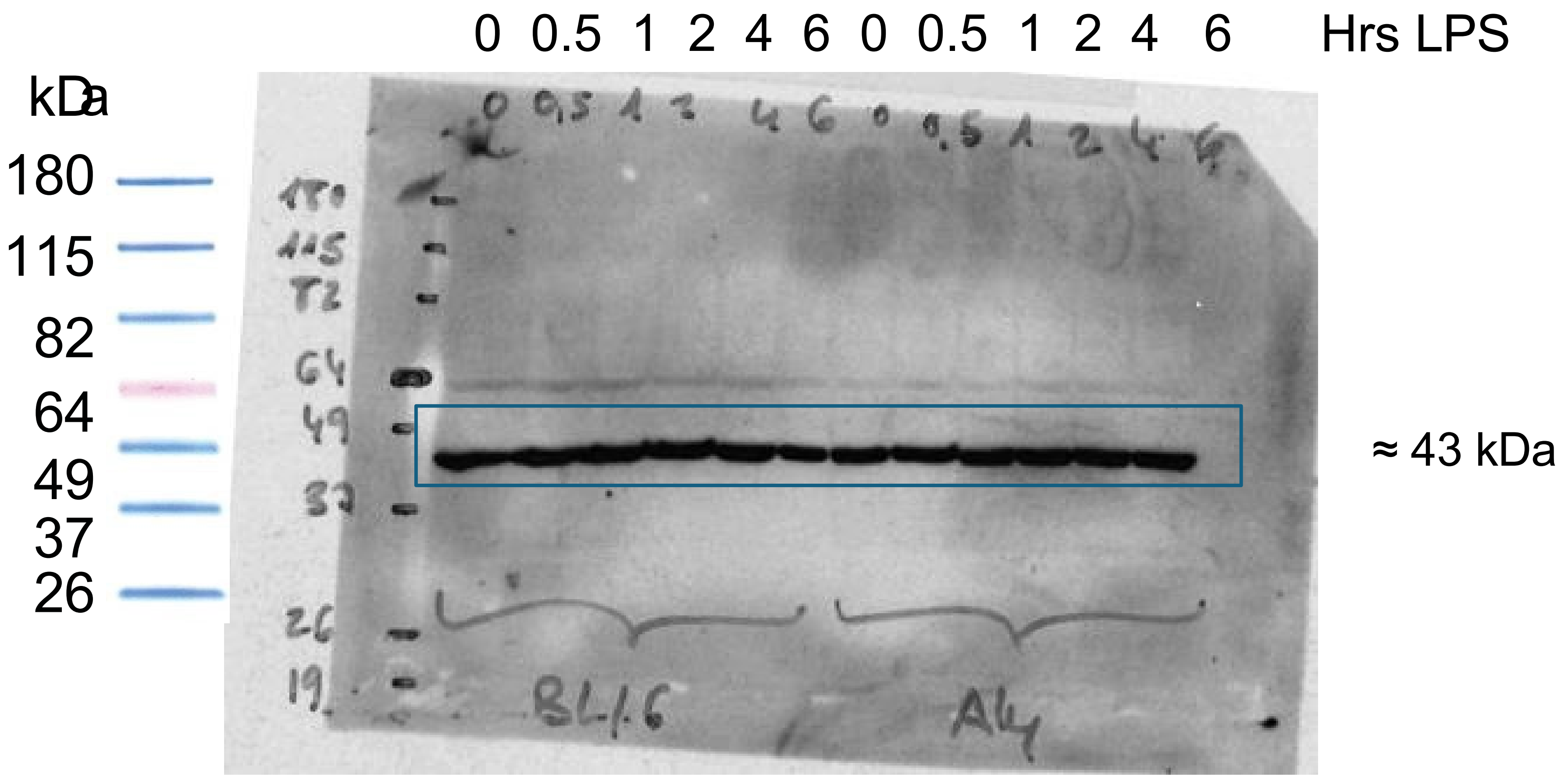

Fig 5 A, lane 6. Cytoplasmic extracts. NIK

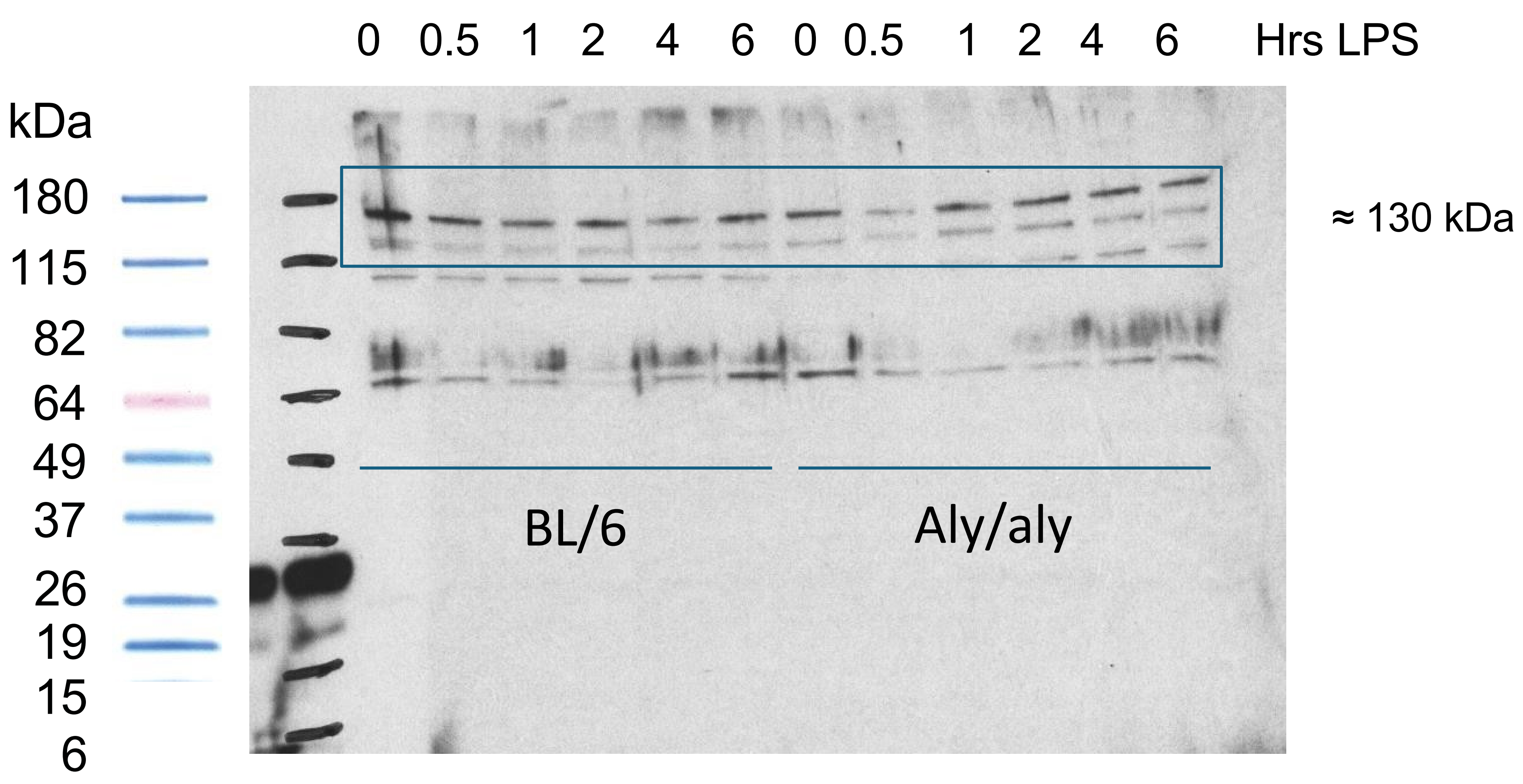

Fig 5 A, lane 7. Cytoplasmic extracts. c-Rel

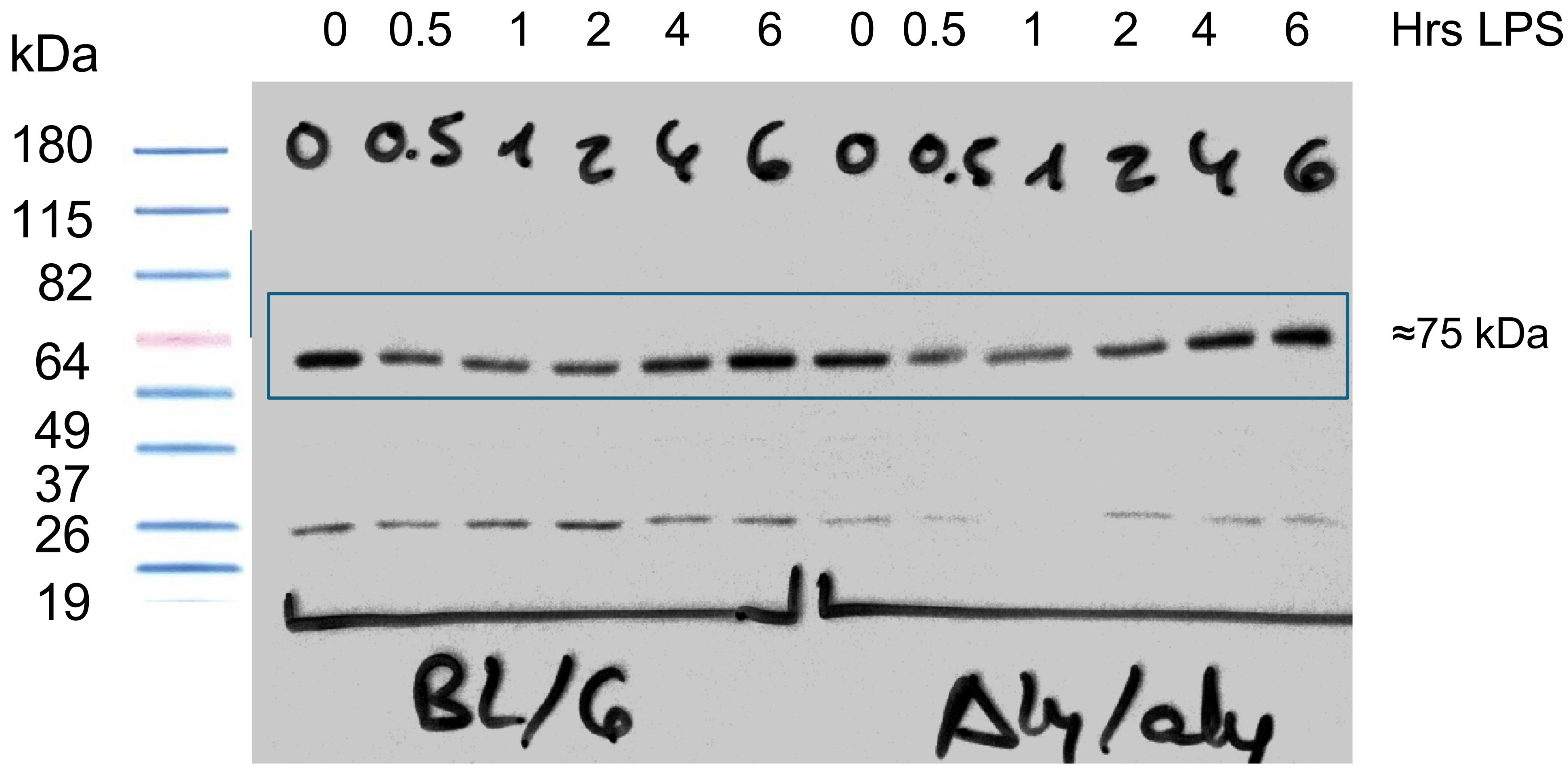

Fig 5 A, lane 8. Cytoplasmic extracts. p65

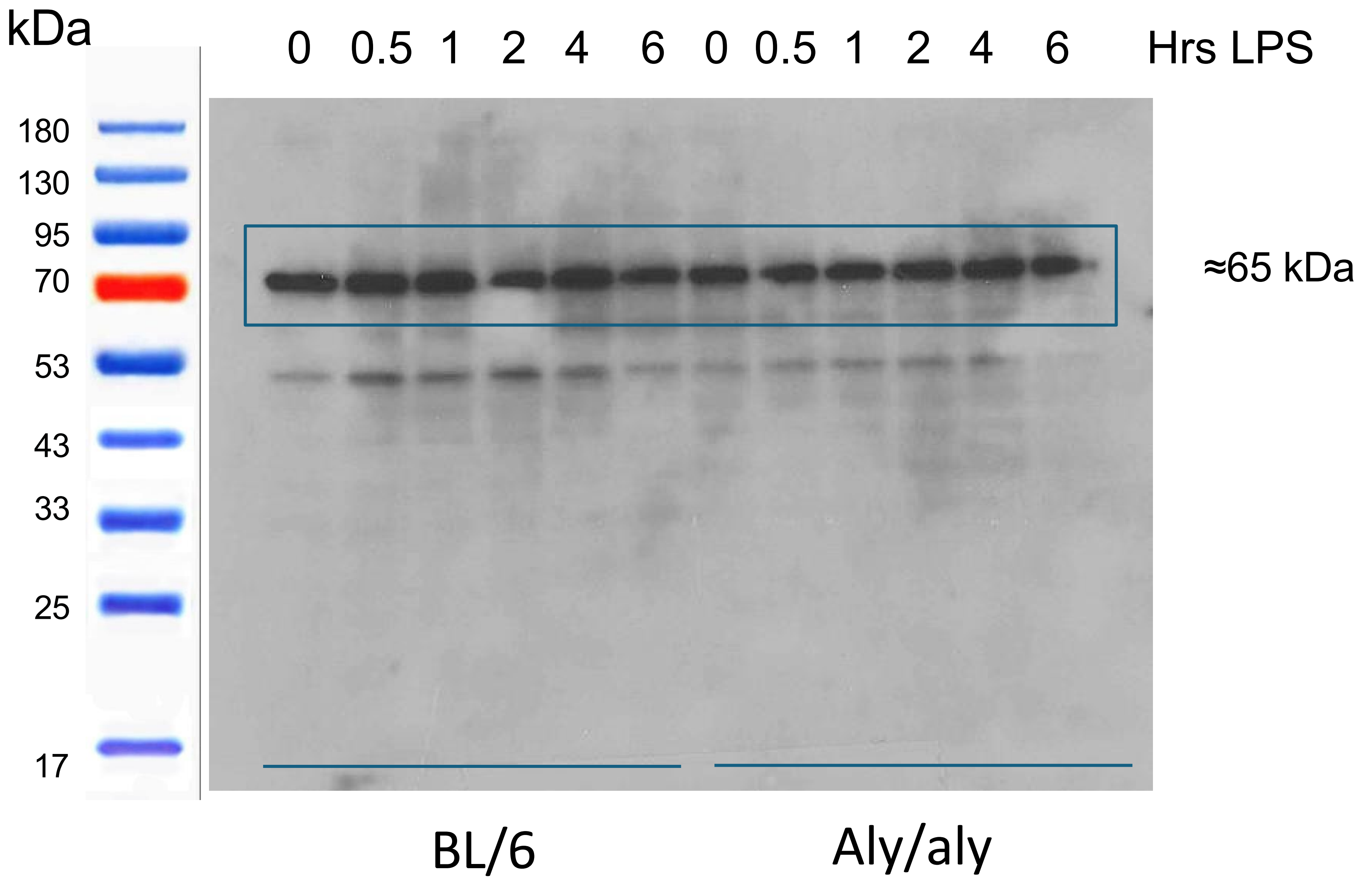

Fig 5 A, lane 9. Cytoplasmic extracts. p50

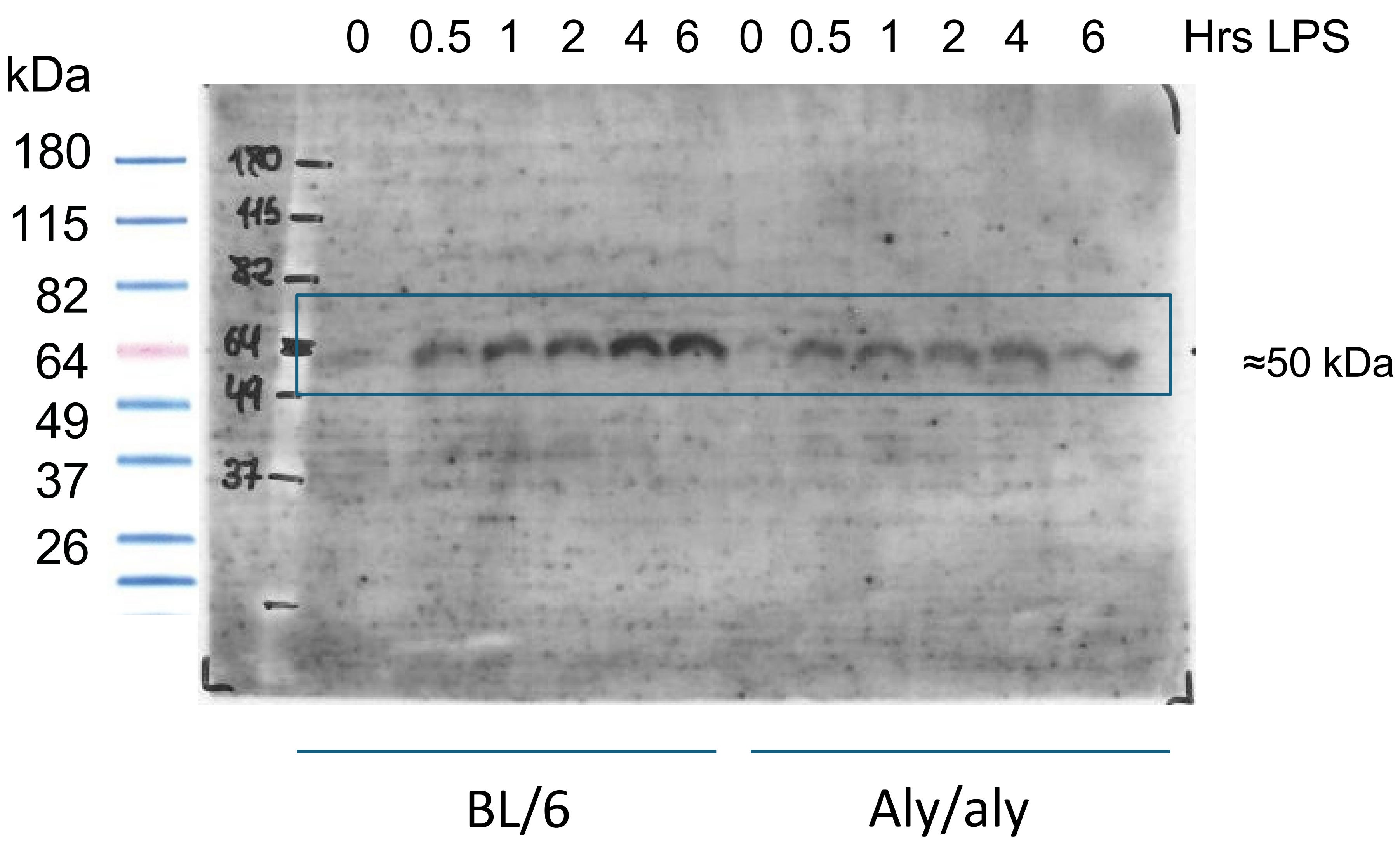

Fig 5 A, lane 10. Cytoplasmic extracts. β-actin

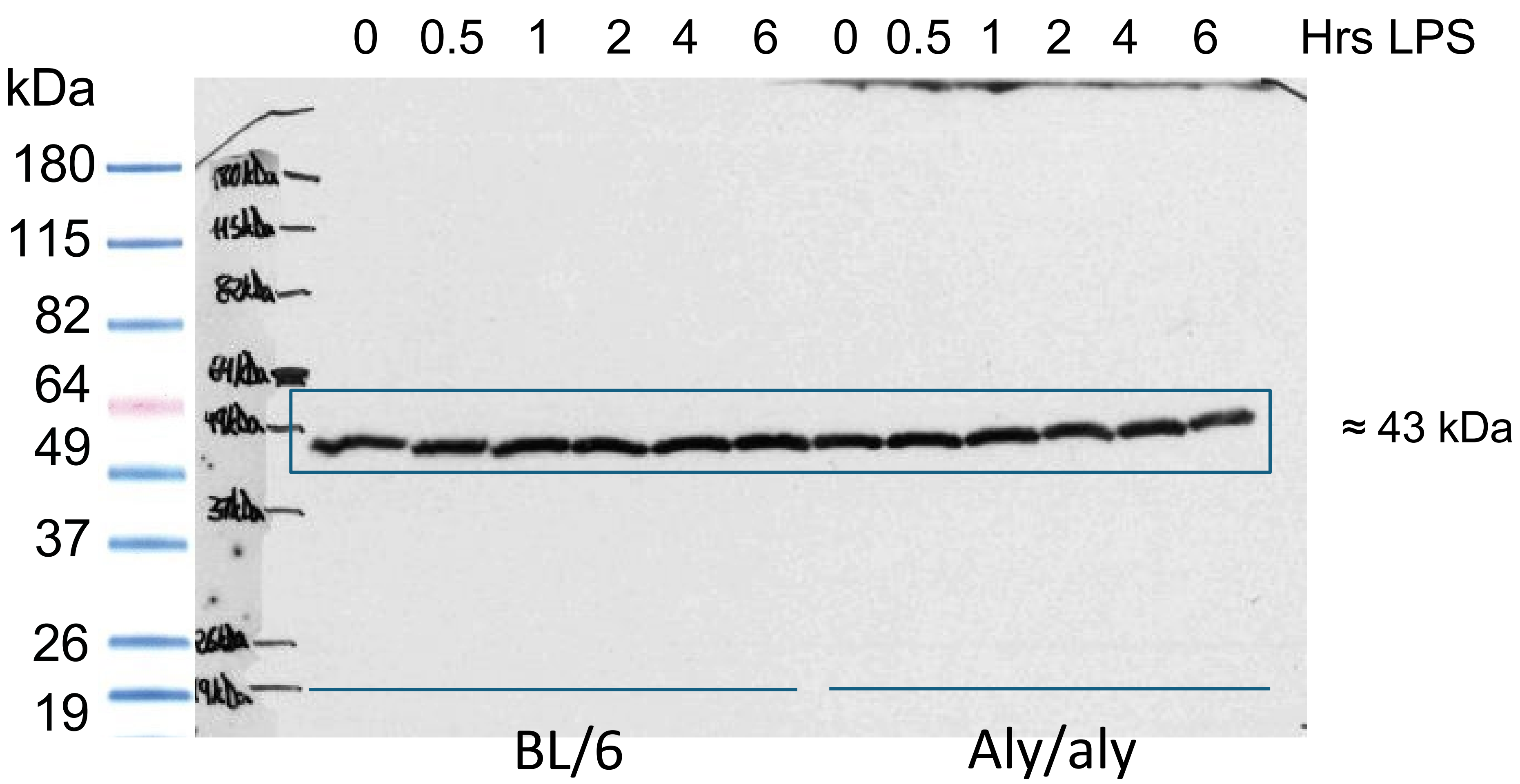

Fig 5 A, lane 11. Nuclear extracts. NIK

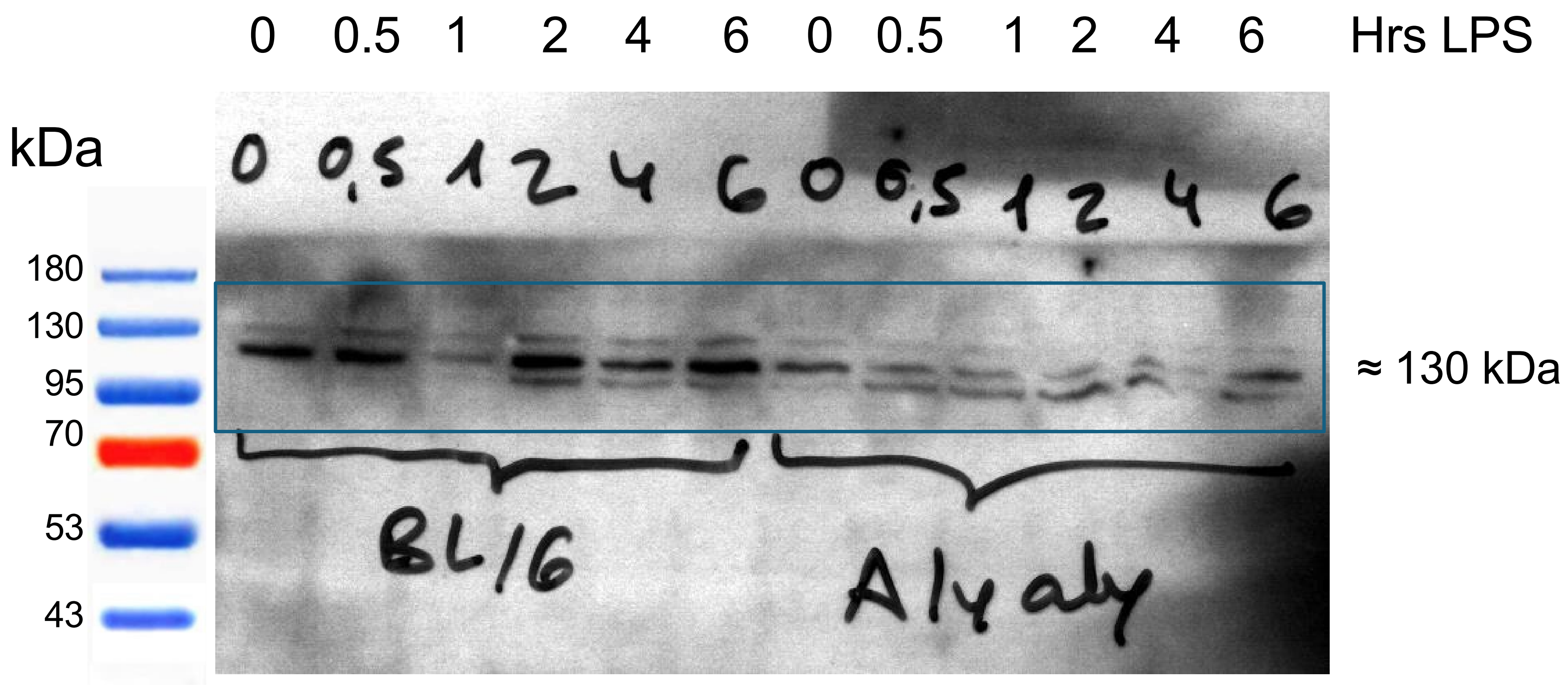

Fig 5 A, lane 12. Nuclear extracts. c-Rel

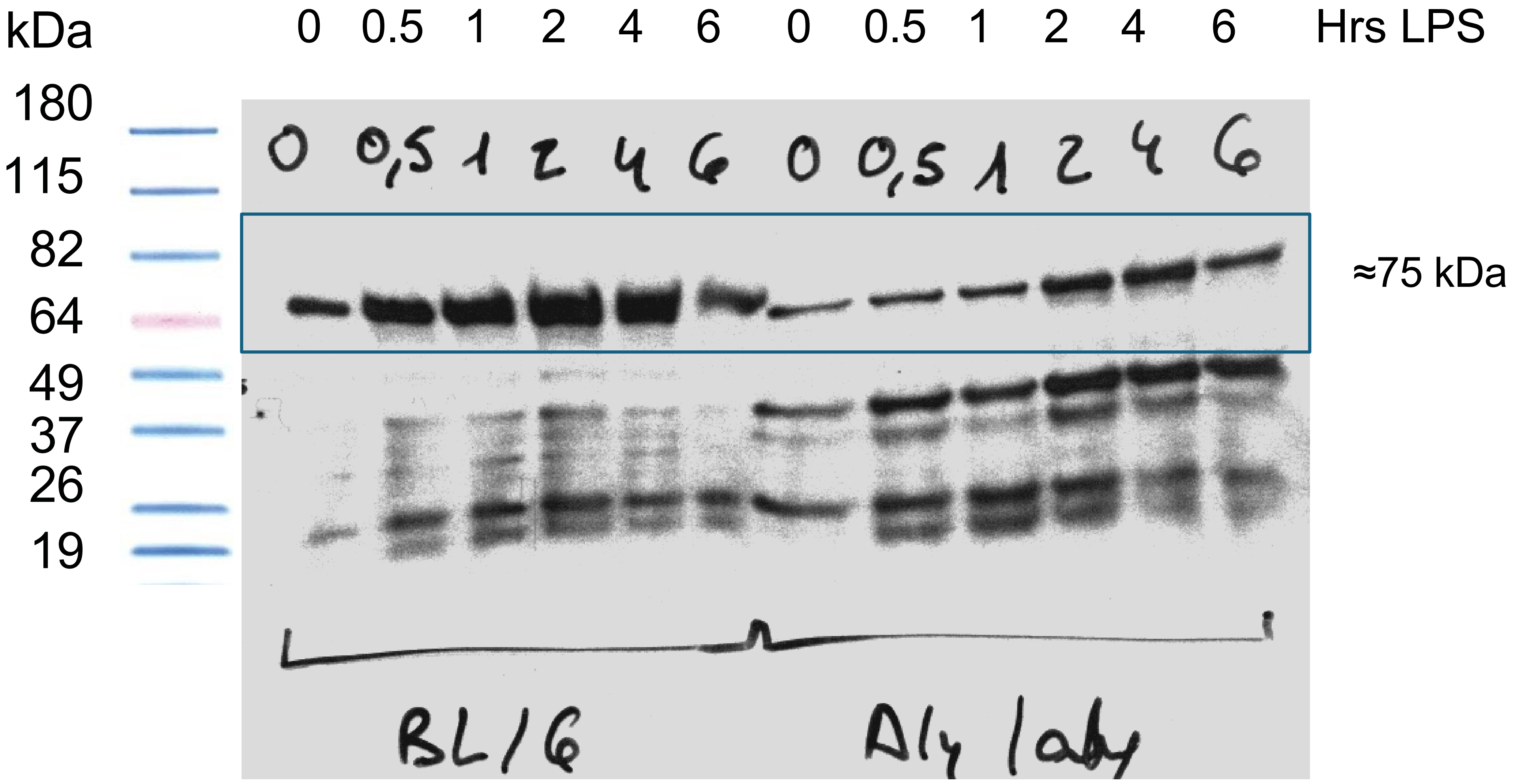

Fig 5 A, lane 13. Nuclear extracts. p65

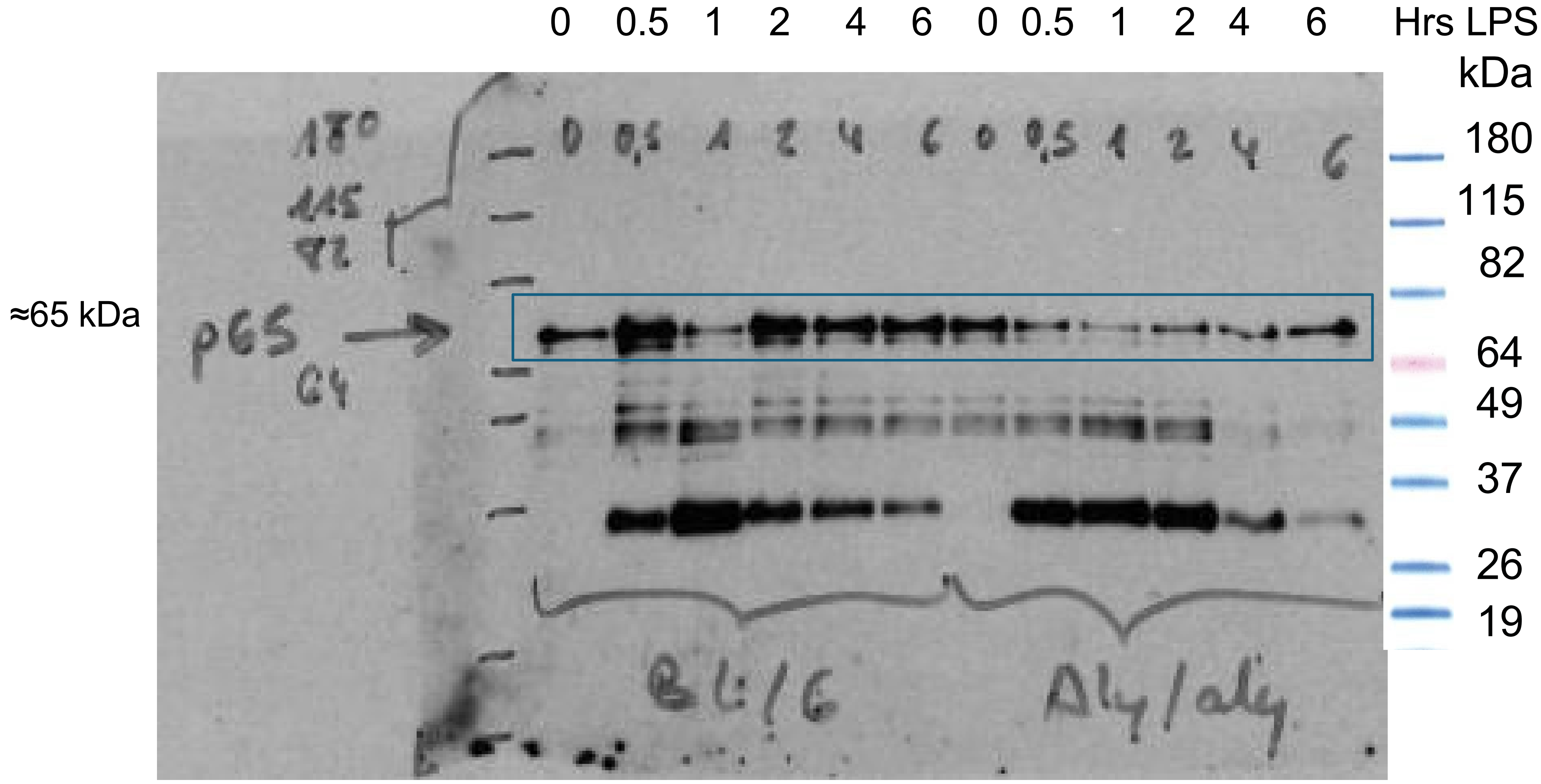

Fig 5 A, lane 14. Nuclear extracts. p50

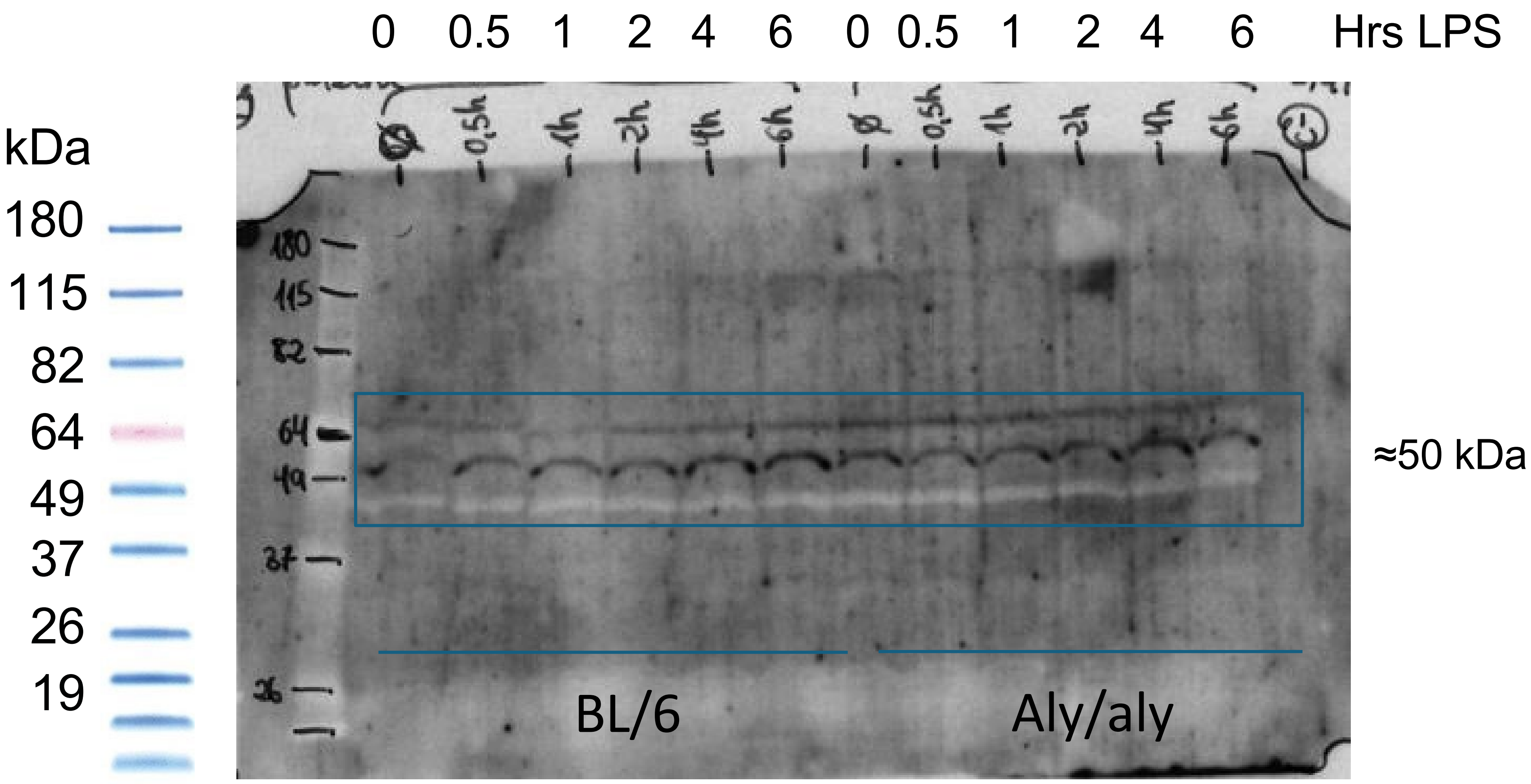

Fig 5 A, lane 15. Nuclear extracts. Nucleolin

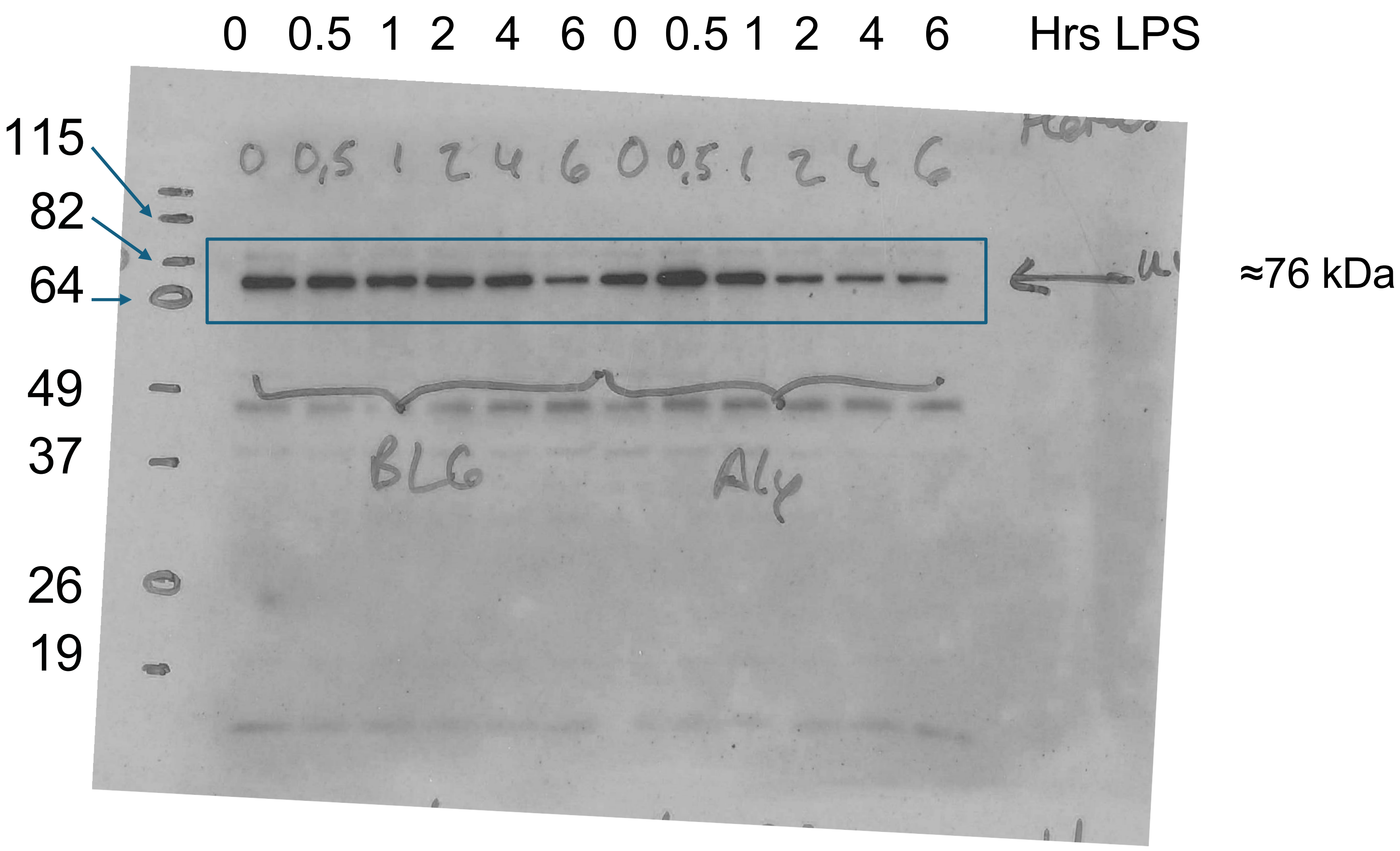

Fig 6 A, lanes 1 and 2

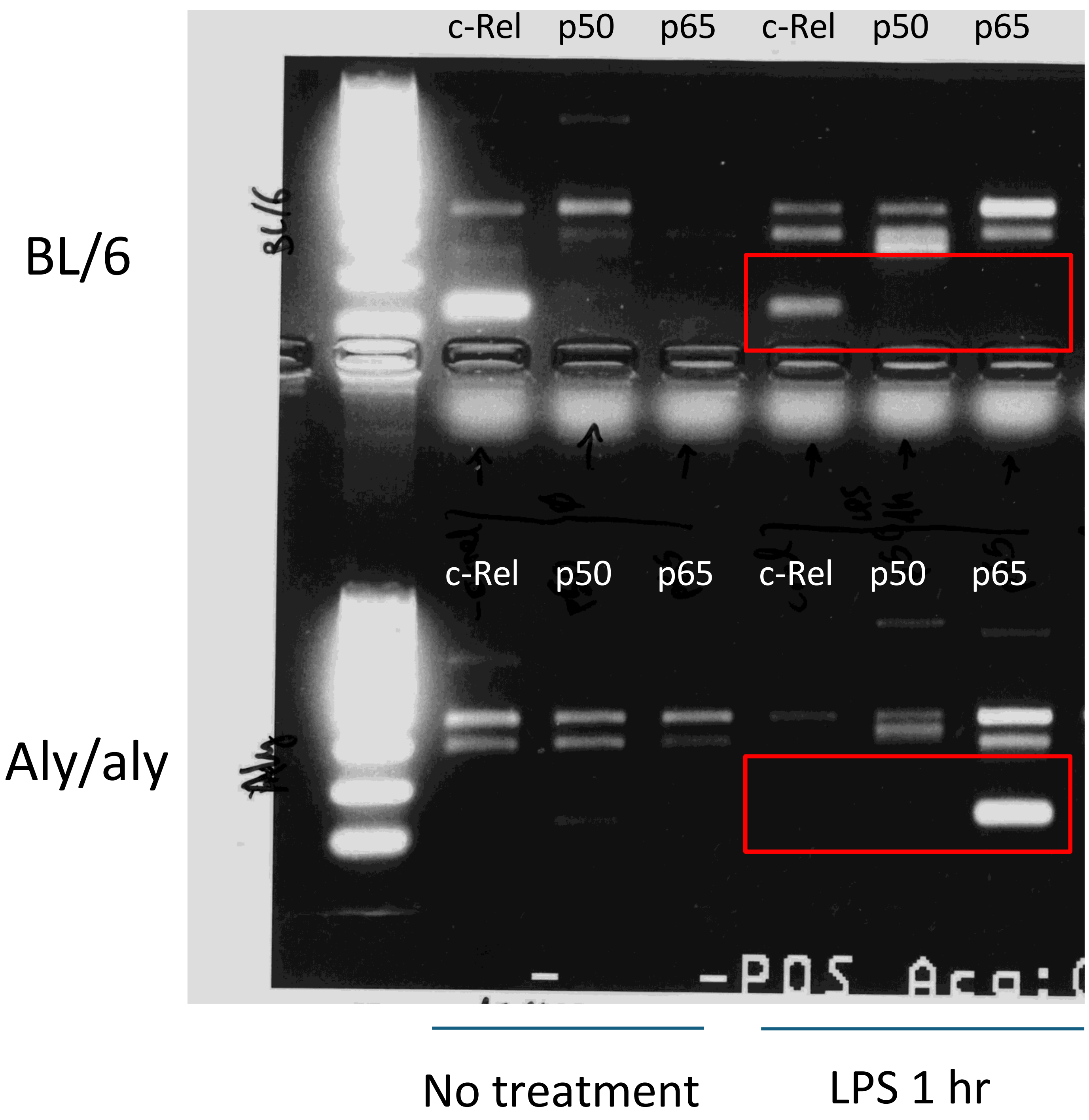

Fig 6 B, lanes 1 and 2

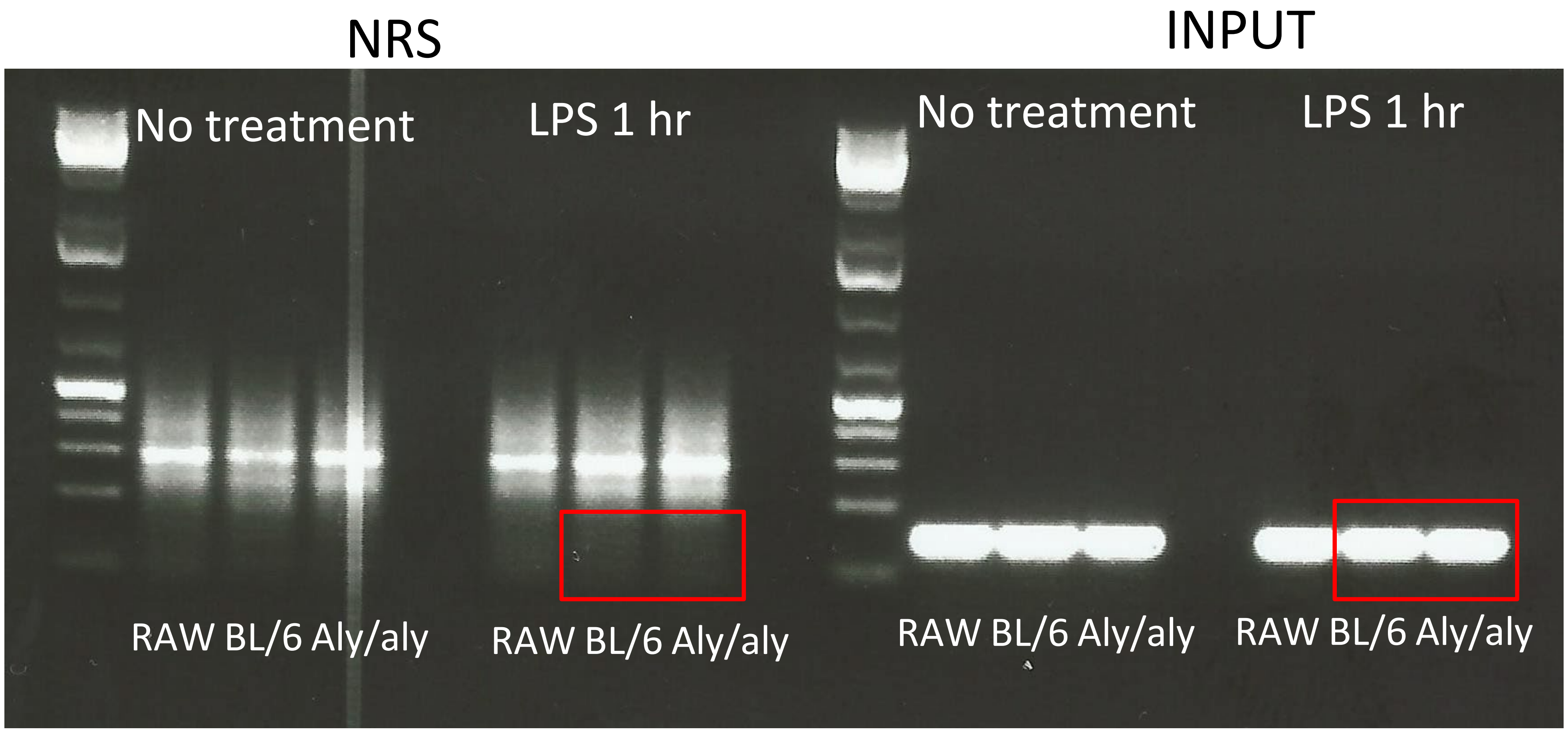

Supplement: Supplementary file 1 [file biology-14-00033-s001.zip › biology-3356810-supplementary.pdf]
